# Mangiferin: An effective agent against human malignancies

**DOI:** 10.1002/fsn3.4434

**Published:** 2024-09-08

**Authors:** Nimra Irshad, Hammad Naeem, Muhammad Shahbaz, Muhammad Imran, Ahmed Mujtaba, Muzzamal Hussain, Waleed Al Abdulmonem, Suliman A. Alsagaby, Tadesse Fenta Yehuala, Mohamed A. Abdelgawad, Mohammed M. Ghoneim, Ehab M. Mostafa, Samy Selim, Soad K. Al Jaouni

**Affiliations:** ^1^ Department of Food Science and Technology Muhammad Nawaz Shareef University of Agriculture, Multan Multan Pakistan; ^2^ Post‐Harvest Research Centre Ayub Agricultural Research Institute, Faisalabad Faisalabad Pakistan; ^3^ Department of Food Science and Technology University of Narowal Narowal Pakistan; ^4^ Department of Food Sciences and Technology, Faculty of Engineering Sciences and Technology Hamdard University Islamabad Campus Islamabad Pakistan; ^5^ Department of Food Sciences Government College University Faisalabad Faisalabad Pakistan; ^6^ Department of Pathology, College of Medicine Qassim University Buraidah Saudi Arabia; ^7^ Department of Medical Laboratory Sciences, College of Applied Medical Sciences Majmaah University AL‐Majmaah Saudi Arabia; ^8^ Faculty of Chemical and Food Engineering, Bahir Dar Institute of Technology Bahir Dar University Bahir dar Ethiopia; ^9^ Department of Pharmaceutical Chemistry, College of Pharmacy Jouf University Sakaka Aljouf Saudi Arabia; ^10^ Department of Pharmacy Practice, College of Pharmacy AlMaarefa University Ad Diriyah Riyadh Saudi Arabia; ^11^ Department of Pharmacognosy, College of Pharmacy Jouf University Sakaka Saudi Arabia; ^12^ Pharmacognosy and Medicinal Plants Department, Faculty of Pharmacy (Boys) Al‐Azhar University Cairo Egypt; ^13^ Department of Clinical Laboratory Sciences, College of Applied Medical Sciences Jouf University Sakaka Saudi Arabia; ^14^ Department of Hematology/Oncology, Yousef Abdulatif Jameel Scientific Chair of Prophetic Medicine Application, Faculty of Medicine King Abdulaziz University Jeddah Saudi Arabia

**Keywords:** anticancer, bioactive compound, chemopreventive, chronic pancreatitis, mangiferin, therapeutic alternative

## Abstract

Mangiferin is a bioactive substance present in high concentration in mangoes and also in some other fruits. Owing to its potential as a chemopreventive and chemotherapeutic agent against several types of cancer, this unique, significant, and well‐researched polyphenol has received a lot of attention recently. It possesses the ability to treat cancers, including rectal cancer, prostate cancer, ovarian cancer, leukemia, gastric cancer, liver cancer, chronic pancreatitis, and lung cancer. It can control/regulate multiple key signaling pathways, such as signal transducer and activator of transcription 3 (STAT3), second mitochondria‐derived activator of caspases/direct inhibitor of apoptosis (IAP)‐binding protein with low propidium iodide (pl) (Smac/DIABLO) nuclear factor kappa B (NF‐κB), phosphatidylinositol 3 kinase/protein 3 kinase (PI3K/Akt), transforming growth factor beta/suppressor of mothers against decapentaplegic (TGF‐β/SMAD), c‐jun N‐terminal kinase/p38 mitogen‐activated protein kinase (JNK/p38‐MAPK), and phosphor‐I kappa B kinase (p‐IκB), which are crucial to the development of cancers. By triggering apoptotic signals and halting the advancement of the cell cycle, it can also prevent some cancer cell types from proliferating and developing. It has been revealed that mangiferin targets a variety of adhesion molecules, cytokines, pro‐inflammatory transcription factors, kinases, chemokines, growth factors, and cell‐cycle proteins. By means of preventing the onset, advancement, and metastasis of cancer, these targets may mediate the chemopreventive and therapeutic effects of mangiferin. Mangiferin has confirmed potential benefits in lung, cervical, breast, brain, and prostate cancers as well as leukemia whether administered alone or in combination with recognized anticancer compounds. More clinical trials and research investigations are required to completely unleash the potential of mangiferin, which may lower the risk of cancer onset and act as a preventive and therapeutic alternative for a number of cancers.

## Graphical abstract

Mangiferin is a bioactive ingredient obtained from mango tree (*Mangifera indica* L.), a plant that has multiple uses in medicine. Mangiferin has confirmed potential benefits in lung, cervical, breast, brain, and prostate cancers as well as leukemia whether administered alone or in combination with recognized anticancer compounds. More clinical trials and research investigations are required to completely unleash the potential of mangiferin, which may lower the risk of cancer onset and act as a preventive and therapeutic alternative for a number of cancers.

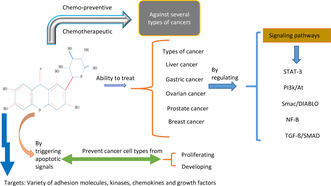

## 1 INTRODUCTION

One of the biggest health issues of the modern period is cancer, which remains currently the leading cause of death followed by heart diseases. Uncontrollably proliferating cells that eventually form tumors are the characteristic features of cancer. Both somatic mutations in upstream cell‐signaling pathways and genetic abnormalities in any gene encoding cell‐cycle proteins can cause it. Every year, millions of patients die from cancer‐related causes, despite the growing availability of traditional cancer treatment alternatives (Schabath & Cote, 2019). With around 10 million deaths from cancer in 2020, it is the world's top cause of death. In 2020, breast cancer accounted for 2.26 million of all new cases, while lung cancer accounted for 2.21 million. Risk factors for cancer and other noncommunicable diseases include smoking, drinking, eating poorly, not exercising, and being around pollutants in the air. *Helicobacter pylori*, human papillomavirus (HPV), hepatitis B, hepatitis C, and Epstein–Barr viruses are among the carcinogenic illnesses that were linked to almost 13% of malignancies diagnosed worldwide in 2018. Furthermore, the incidence rates in transitional economies are 200%–300% higher than in impoverished countries for several of the most common kinds of cancer. In developing nations, the incidence of cancer linked to infections is higher, and the cost of cancer connected with Western lifestyle choices is rising (Cox, 2021).

Bioactive substances derived from plants, which are created by them for defense, are known as phytochemicals. More than a thousand phytochemicals have been identified, and they can be obtained from a variety of foods, including whole grains, fruits, vegetables, nuts, and herbs. Numerous plants, fruits, vegetables, and leaves contain phytochemicals called flavonoids, which may have uses in medicinal chemistry. Numerous health advantages of flavonoids include their antiviral, anticancer, antioxidant, and anti‐inflammatory qualities (Ranjan et al., 2019).

A variety of plants also contain the chemical known as mangiferin, however it is mostly derived from the Indian delicacy known as “*Mangifera indica*,” the “king of fruits.” Regarded as one of the primary active ingredients in over 40 polyherbal preparations, mangiferin is utilized in traditional Chinese medicine (TCM). It was first discovered in plants belonging to 16 different families, including the Anacardiaceae, Gentianaceae, and Iridaceae. However, it was initially mostly isolated from *Mangifera indica*. The stem, fruit, and leaves of the plants have all been divided from it (Morozkina et al., 2021). Mangiferin's structure is made up of one main glycosidic hydroxyl group, one lactonic carbonyl group, two aromatic rings, and nonaromatic secondary hydroxyl groups. It has therapeutic and biological potential because of the functional groups. It is the base of two recognized Cuban inventions: “Alpizarin—an antiviral drug” and “Vimang—anti‐inflammatory and immunomodulatory” (Dutta et al., 2023).

**FIGURE 1:**
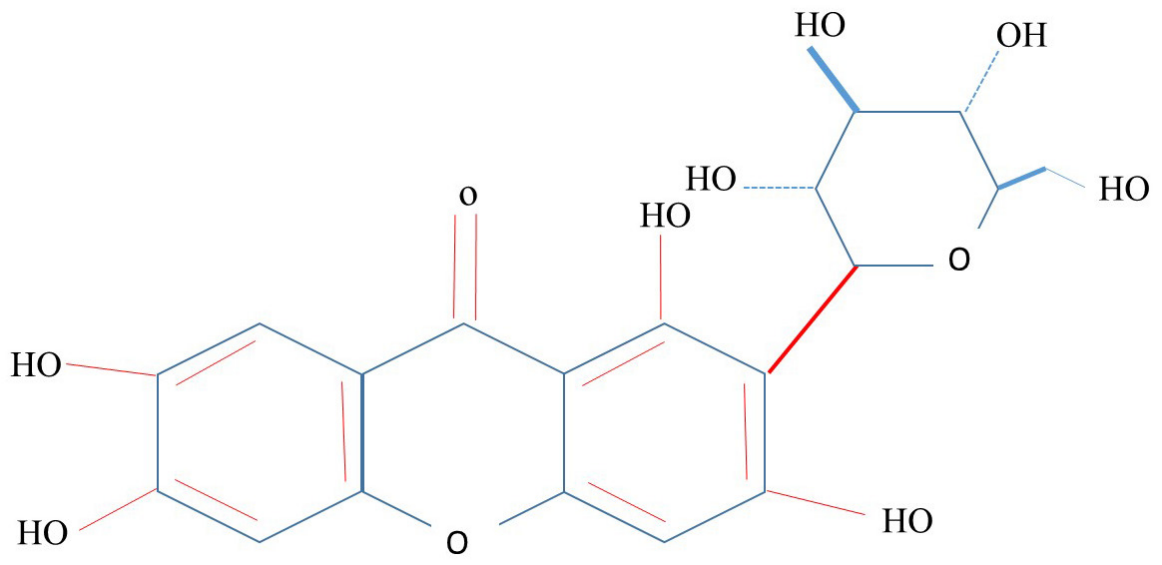
Chemical structure of mangiferin.

Mangiferin, (C_19_H_18_O_11_), is an active phytochemical and due to its low solubility at room temperature and its strong solubility in hot water it is simply extracted into infusions. The health benefits of these products are mostly related to the antioxidant qualities that mangiferin has. It displays a minor solubility in water and ethanol, while it remains amorphous in some nonpolar liquids like diethyl ether. Its solubility in water is 0.111 mg/mL. Some countries, including Cuba, China, and India, have long grown and actively employed mangiferin‐rich plants in conventional medicine to cure a diversity of ailments, for example various infections, diabetes, cardiovascular disease, and cancer (Akter et al., 2022). Figure 1 shows the chemical structure of mangiferin.

## 2 BIOAVAILABILITY AND PHARMACOKINETICS OF MANGIFERIN

The active ingredient in therapeutic herbs, mangiferin has low hydrophilicity and lipophilicity. It can be characterized as a chemical in class IV (Low Solubility–Low Permeability) of the Biopharmaceutics Classification System (BCS) because of its low water solubility and poor intestinal penetrability. Specially, in terms of solubility. In phosphate buffer (pH 6.8), mangiferin has an oil–water partition coefficient of 0.15, suggesting a low lipophilicity that may result in poor intestinal membrane permeability (Di Lorenzo et al., 2021). In another research study, blood samples were analyzed at 0, 0.5, 1, 2, 3, 4, 5, 6, 8, 10, and 12 h after oral administration of three different mangiferin solutions at dosages of 25, 50, and 100 mg/kg to test subjects (*n* = 6 rats in each group). This encouraged researchers to determine the oral bioavailability of mangiferin. The findings revealed that the absolute bioavailability of mangiferin at the tested levels is less than 1%, declining as one increases the dose. Consequently, despite the range of pharmacological effects, mangiferin's low bioavailability has severely restricted its clinical use (Mei et al., 2023).

Researchers have explored improvements in membrane permeability in order to enhance mangiferin's absorption in the gastrointestinal system. The pharmacokinetic characteristics demonstrated a four‐ to sevenfold increase in mangiferin oral absorption when absorption enhancers were present. The chosen enhancers of absorption were Carbopol 974P (100 mg/kg) and sodium deoxycholate (90 mg/kg), which were employed following the oral administration of 30 mg/kg of mangiferin. Sodium deoxycholate appeared to boost absorption because it changed the compactness within the lecithin bilayer of lecithin molecules, which increased phospholipid mobility and permitted mangiferin to pass through the bilayer and be absorbed. Furthermore, because sodium deoxycholate chelates Ca_2_^+^, it increases paracellular permeability, which disrupts intracellular tight junctions. When mangiferin and the bioadhesion polymer Carbopol 974P (100 mg/kg) were combined, the mucoadhesion of Carbopol 974P allowed mangiferin to be localized at the site of absorption for a longer contact period, increasing the oral bioavailability. The complex‐forming calcium‐binding protein Carbopol 974P also enhanced paracellular mangiferin's penetration. It has also been reported that complexing mangiferin with soy phospholipids enhances its bioavailability as well as its in vivo antioxidant and hepatoprotective properties. The mangiferin–phospholipid complex, which was generated at a molar ratio of 1:2, was confirmed by using differential Fourier transform infrared (FT‐IR) spectroscopy and thermal analysis (Zivkovic et al., 2023).

In terms of pharmacokinetic properties, the mangiferin–phospholipid complex showed an increase in both Cmax and Tmax. Cmax increased from 0.34 ± 0.02 μg/mL to 1.22 ± 0.14 μg/mL. It was discovered that the complex's area under the curve (AUC) (0‐t) was 9.75 times greater than that of pure mangiferin. Eventually, mangiferin–phospholipid complex demonstrated more antioxidant activity than mangiferin alone when given to experimental animals (60 mg/kg b.w.). Antioxidant enzyme levels (reduced glutathione (GSH), glutathione peroxidase (GPX), glutathione S‐transferase (GST), glutathione reductase (GR), superoxide dismutase (SOD), and catalase (CAT)) were able to be restored to normal by the complex. Furthermore, compared to mangiferin alone, the mangiferin–phospholipid complex demonstrated greater hepatoprotective efficacy against lipid peroxidation by reducing thiobarbituric acid reactive compounds. In recent times, magnetite microspheres have been employed as a medication administration method for mangiferin because of their capability to improve the solubility in water. Because of their ability to use a magnetic field to increase medication concentration at the target region, magnetic polymeric microspheres have been exposed to be a suitable controlled release and tailored medication delivery methods for the management of cancer. Solvent diffusion was used to produce magnetic poly (ε‐caprolactone)–poly (ethyleneglycol)–poly (ε‐caprolactone) (PCEC) microparticles loaded with mangiferin. The nanoparticles had a controlled delivery and an average diameter of 200 nm, making them nearly spherical, with an encapsulation efficiency of roughly 50% (Telange et al., 2021). To determine whether the anticancer potential of mangiferin was preserved, the in vitro cytotoxic effects of mangiferin alone and mangiferin‐loaded magnetic PCEC microparticles were examined against the proliferation of different breast, lung, and cervical carcinoma cells. Mangiferin‐loaded magnetic PCEC microparticles were found to have antiproliferative activity that was dose‐dependent and larger than that of mangiferin alone, suggesting that magnetite PCEC microspheres may have vast potential as an effective mangiferin carrier (Liu et al., 2020).

## 3 ANTIOXIDANT STATUS

The ability to counteract excess oxygen free radicals (OFRs) produced by environmental factors is recognized as antioxidant potential. Rat hepatocytes or their subcellular fractions (microsomes and mitochondria) include the cell model system that is commonly hired to evaluate the antioxidant activity of mangiferin (Szmidt et al., 2020). Oxygen is necessary for aerobic life. Oxidative stress can come from both endogenous and external sources in aerobic life. Oxygen free radicals (OFRs) cause widespread cell damage, in spite of antioxidant defense mechanisms. Cancer can develop more quickly when OFR‐related lesions do not result in cell death. It is often believed that oxidative DNA damage causes mutagenesis frequently in healthy human cells (Zou et al., 2017). Numerous studies have indicated that OFR plays crucial roles in the growth of tumor clones and their development into malignant entities. It has been established that malignant melanocyte transformation and oxidative stress (OS) are related. Numerous diseases, including diabetes mellitus, cardiovascular disease, and neurodegenerative illness, have been related to oxidative stress, which is characterized as a relative excess of reactive oxygen species (ROS) as compared with antioxidants (Wang et al., 2021).

Although ROS are pro‐tumorigenic, elevated ROS levels are lethal, and cancer cells have abnormal redox homeostasis (Klaunig, 2018). Tumor cells specifically produce a lot of reactive oxygen species (ROS) during their hyperproliferation, but they are adapted to survive in environments where this oxidative burden shifts the redox balance away from a reduced state. Tumor cells accomplish this by boosting their antioxidant status to maximize ROS‐driven proliferation while simultaneously avoiding ROS thresholds that will cause senescence, apoptosis, or ferroptosis (Jelic et al., 2021).

Studies in the laboratory and on epidemiology have demonstrated that when the excess oxidants are not counterbalanced by antioxidant defense and/or DNA repair mechanisms, the overproduction of reactive oxygen and nitrogen species can result in an alteration of intracellular homeostasis and damage to all the major components of the cell. By changing the expression of genes linked to cancer and resulting in mutation and transformation, chronic oxidative stress can promote the development of cancer. Reactive oxygen and nitrogen species are now widely accepted to play a role in the initiation and advancement of a number of human malignancies, including those of the breast, prostate, colorectal, gynecological, cervical, eye, skin, leukemia, and stomach (Kruk & Aboul‐Enein, 2017).

Enzymes that are known to provide antioxidant defenses include GPX, catalase (CAT), glutathione reductase (GR), and superoxide dismutase (SOD). The common antioxidants swallowed as part of the diet with potential health advantages are nonenzymatic antioxidants, which are represented by a number of dietary components, including Vitamin A, C, E and phenolic compounds. The antioxidant activity of mangiferin was assessed using a range of techniques, such as the 1,1‐diphenyl‐2‐picrylhydrazyl (DPPH) radical test and enzymatic and nonenzymatic lipid peroxidation assays using rat liver microsomes. In the in vitro DPPH and nonenzymatic lipid peroxidation tests, mangiferin demonstrated antiradical action at a 200 μM dosage. Mangiferin's antioxidant activity was most likely resultant from its capacity to hunt free radicals, as it did not prevent the lipid peroxidation that was produced by enzymes (Morozkina et al., 2021). The ability of *Mangifera indica* stem bark extract (MSBE) to scavenge free radicals implicated in rat liver microsome lipid peroxidation formed by adenosine diphosphate/iron/nicotinamide adenine dinucleotide phosphate (ADP/Fe/NADPH) has been established by researchers as the source of MSBE's antioxidant activity. They investigated the hydroxyl‐mediated oxidation of bovine serum albumin (BSA) and the formation of carbonyl groups using the same hepatic microsome technique. With the exception of the NADPH‐dependent cytochrome P450 (CYP450) reductase activity measurement, where MSBE had no influence on the oxidation rate of NADPH, all assays showed that it was active at very low concentrations (0.0025% w/v (percentage weight by volume)). Another study established that MSBE and mangiferin, at dosages ranging from 10 to 473 μM, may prevent lipid peroxidation and the ensuing cell damage because they can scavenge free radicals, primarily lipoperoxyl and alkoxyl radicals, and create stable iron complexes. Furthermore, a different study revealed that at doses ranging from 50 to 250 μg/mL, the MSBE and mangiferin reduced the activity of certain cytochrome P450 enzymes, changing the levels of the messenger RNA (mRNA) that codes for a few of these enzymes (Cheng et al., 2021). Other cell model systems that resemble the central nervous system (CNS) have also been used to assess mangiferin's antioxidant activities. Oligodendrocytes and cortical neurons were partially shielded from mild damage by micromolar concentrations of mangiferin (10–100 μM). This damage was caused by α‐amino‐3‐hydroxy‐5‐methyl‐4‐isoxazolepropionic acid (AMPA) receptors, which increased the cytoplasmic concentration of Ca^2+^ and increased the generation of ROS by the mitochondria. Mangiferin once more showed the ability to scavenge oxygen radicals and decrease intracellular Ca^2+^ excess upon AMPA receptor activation (Telange et al., 2021).

In the murine neuroblastoma cell line N2A, where oxidative stress is generated by the active metabolite of 1‐methyl‐4‐phenyl‐1,2,3,6‐tetrahydropyridine (MPTP), 1‐methyl‐4‐phenyl‐pyridinium ion (MPP^+^), the antioxidant activity of mangiferin was also demonstrated. Researchers showed that treatment with mangiferin (6.25–25 μg/mL) protected N2A cells against MPP^+^‐induced cytotoxicity through the downregulation of mRNA coding for two essential antioxidant enzymes, superoxide dismutase 1 (SOD1) and catalase, and the restoration of glutathione (GSH) level (Jayasuriya & Ramkumar, 2022).

Similar outcomes were obtained when glutamic acid was used to cause neurotoxicity in rat cortical neurons. In this instance, a concentration of 30 μM of mangiferin provided the greatest defense against the oxidative stress brought on by glutamate. Experimental animals given doses of 10, 20, and 40 mg/kg b.w. (milligrams per kilogram of body weight) for 14 days prior to therapy have demonstrated the neuroprotective effectiveness of mangiferin against the Parkinson's disease (PD) model in mice. The findings demonstrated that mangiferin inhibited the behavioral abnormalities, oxidative stress, apoptosis, dopaminergic neuronal degeneration, and dopamine depletion caused by MPTP, indicating that mangiferin's strong antioxidant and anti‐apoptotic qualities were the cause of neuroprotection (Gold‐Smith et al., 2016). Comparable results were observed in both in vitro (100 nM) and in vivo (10 mg/kg b.w.) ischemia models; in the former, the compound decreased receptor‐mediated calcium influx, oxidative stress, and apoptosis in neuronal cultures where glutamic acid‐induced cell death occurred. It also decreased the production of ROS and neuronal loss in the hippocampal cornu ammonis (CA1) region caused by transient forebrain ischemia in rats. It was demonstrated by researchers that mangiferin and MSBE both prevented rat macrophages from producing ROS and engaging in phagocytic functions. In actuality, mangiferin at dosages ranging from 50 to 250 mg/kg decreased the number of macrophages in peritoneal exudate after intraperitoneal injection of thioglycollate 5 days prior to intraperitoneal administration. Furthermore, at doses ranging from 0.1 to 100 μg/mL, there was a decrease in the generation of nitric oxide (NO), the extracellular production of ROS, and the phagocytosis of yeast cells by resident peritoneal and thioglycollate‐elicited macrophages. Results of the study validated that MSBE and mangiferin may be helpful in the management of illnesses with immunopathological symptoms (Samadarsi & Dutta, 2020).

**FIGURE 2:**
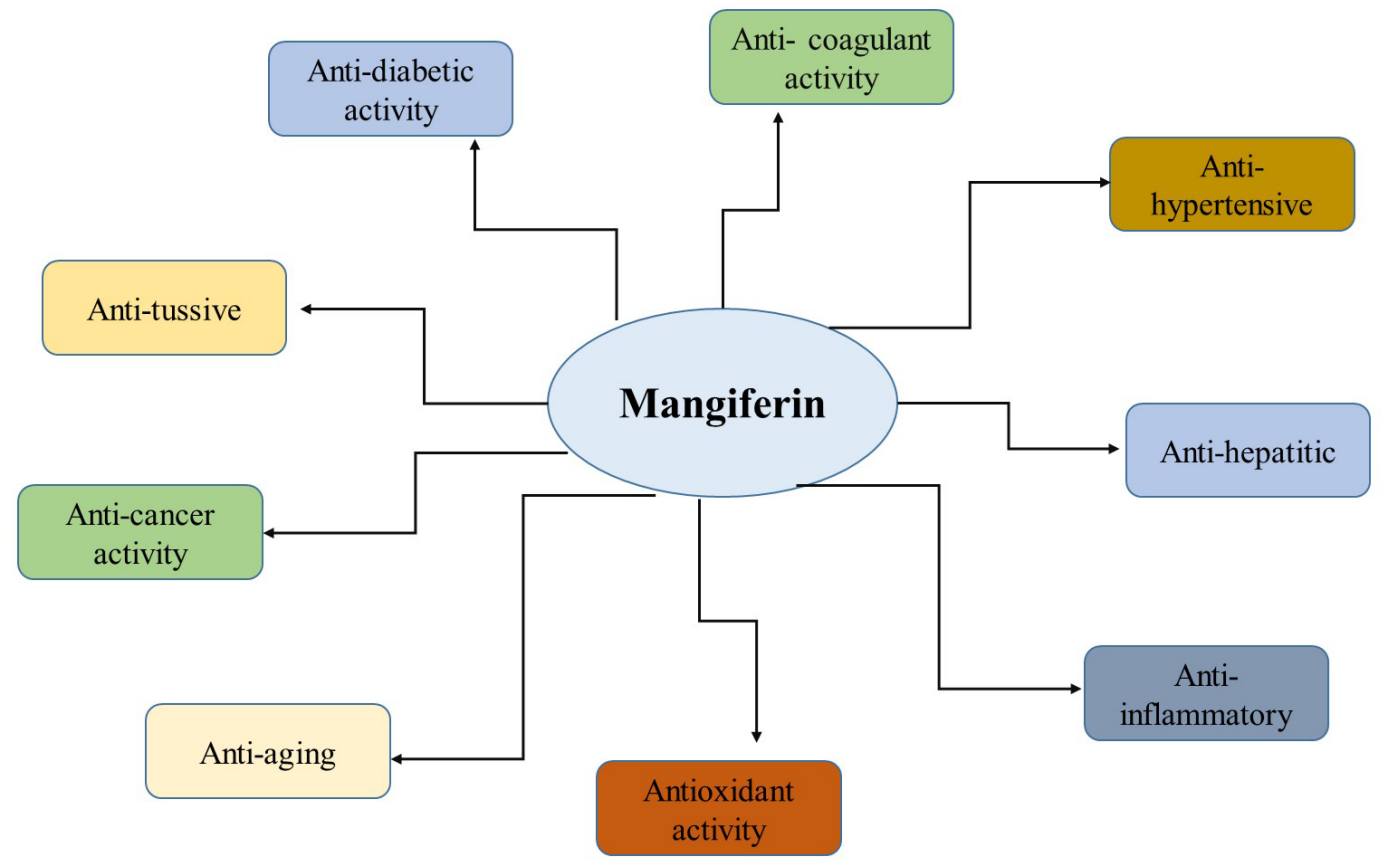
Health benefits of mangiferin.

Researchers observed how mangiferin endangered rats from isoproterenol (ISPH)‐induced myocardial infarction (MI). The elevated activities of lactate dehydrogenase (LDH) and serum creatine phosphokinase isoenzymes (CK‐MB) as well as the rise in uric acid levels and fall in plasma iron binding capacity were used to analyze myocardial damage. The antioxidant enzyme activities (i.e., SOD, CAT, GPX, and glutathione S‐transferase (GST) and glutathione reductase activities) and nonenzymatic antioxidant levels (i.e., ceruloplasmin, Vitamin C, Vitamin E, and GSH levels) were evaluated after mangiferin (100 mg/kg b.w.) was intraperitoneally given to MI rats (i.e., rats suffering from MI) (i.e., rats suffering from MI) for 28 days as a pretreatment. The outcomes demonstrated that mangiferin can restore antioxidant levels and enzymatic activities, indicating that mangiferin may be able to prevent cardiovascular disorders by acting as an antioxidant. Furthermore, 7 days before and 7 days after rats were given hazardous doses of iron–dextran, MSBE and mangiferin pretreatment at doses of 50, 100, 250, and 40 mg/kg b.w., respectively, were able to lower lipid peroxidation and restore antioxidant enzymes. Since these first investigations, numerous more experimental animal model systems have been used to show the in vivo protective action of mangiferin. Furthermore, mangiferin demonstrated protective activity in rats where doxorubicin (DOX) had caused cardiac toxicity at doses ranging from 50 to 100 mg/kg b.w. and in mice where carbon tetrachloride (CCl_4_) had caused hepatic toxicity at a dose of 100 mg/kg b.w (Sguizzato et al., 2021). Figure 2 highlights the health benefits of mangiferin.

## 4 BREAST CANCER

The most common cancer in US women is breast cancer. Not only is it the leading cause of cancer death for women, but it also ranks second in terms of cancer deaths among all women, second only to lung cancer (Koh & Kim, 2019). Because breast cancer is linked to modifiable risk factors, such as being overweight, not exercising, and drinking alcohol, it is believed that 30% of cases can be avoided (Fahad Ullah, 2019). Along with improvements in therapy, mammography screening, or secondary prevention, can also avert death and has been associated with notable failures in the death percentage from carcinoma of breast. In another study, the proliferation of Michigan Cancer Foundation‐7 (MCF‐7) breast cancer cells was halted time‐dependently by mangiferin's suppression of the cyclin‐dependent kinase 1 (CDK1)–cyclin Bl signaling pathway and subsequent G2/M phase cell‐cycle arrest. By inhibiting the protein kinase C (PKC)–nuclear factor kappa B (NF‐κB) pathway, it caused apoptotic cell death. Trials were conducted in vivo against estrogen receptor (ER)‐negative breast cancer using the xenograft mice model and an oral dosage of 100 mg/kg. The amount of mitochondrial cytochrome C (Cyt C) was reduced, indicating that apoptosis can be lessened over the mitochondrial alleyway under mangiferin direction. Additionally, this pathway promoted the overexpression of caspase as well as the downregulation of procaspase appearance (Yap et al., 2021).

Mangiferin decreased tumor volume in C57BL/6J mice by 89.4% when given at a dose of 100 mg/kg, and this reduction was similar to the 91.5% impact of the chemotherapy medication cisplatin. This dosage of mangiferin prolonged the lives of those animals that were treated with it (Zhang et al., 2020). Mangiferin reduced cell survival, reduced matrix metalloproteinase‐ (MMP‐7 and ‐9) synthesis, restricted metastasis, blocked the β‐catenin pathway, and reversed epithelial–mesenchymal transition (EMT) in breast cancer cell lines. In addition to meaningfully reducing tumor proliferation, it also increases apoptosis, weight, and volume. It also modifies angiogenesis and lowers the expression levels of vimentin, MMP‐9, MMP‐7, and β‐catenin activity in a xenograft rat while raising the expression of E‐cadherin. It upsets NF‐κB/DNA binding, inhibitor of nuclear factor kappa B kinase (IκB) degradation, and NF‐κB translocation, to prevent the activation of classical NF‐κB by IκB kinases (IKK) α/β in the cell line of triple‐negative breast cancer (TNBC) MDA‐MB‐231. Furthermore, mangiferin suppresses other NF‐κB pathways: c‐Jun N‐terminal kinases (JNK) 1/2, mitogen‐activated protein kinase kinase (MEK1), p_90_ ribosomal s6 kinase, and mitogen‐ and stress‐activated protein kinase 1. These pathways are implicated in cancer cell survival and showed resistance to treatment. When tumor necrosis factor alpha (TNF‐α) is activated, both Vimang and mangiferin can decrease the construction of interleukin‐6 (IL‐6) and interleukin‐8 (IL‐8), which in turn can lessen the inflammatory response (interleukin‐6 and IL‐8). When compared to the corresponding vehicle‐treated control groups, mangiferin caused a dose‐responsive reduction in the proliferation of MDA‐MB‐231 and MCF‐7 tumor cells after 4 days of therapy. It was revealed that the MCF‐7 cells and MDA‐MB‐231 needed a 10 μM IC50 (half‐maximal inhibitory concentration) dose for mangiferin. Mangiferin caused a dose‐responsive decrease in the viability of MDA‐MB‐231 and MCF‐7 breast cancer cells during a 24‐h treatment period when compared to control cells (Deng et al., 2018).

The in vivo efficacy of isomangiferin was examined using a mouse model of human breast cancer xenograft. Researchers observed the fundamental process in vitro and isomangiferin‐inhibitory effect on breast cancer cells. Isomangiferin therapy condensed proliferation, invasion, migration, and cancer cell adhesion in vitro. Isomangiferin reserve of angiogenesis and its influence on the growth of breast cancer were well coordinated. Moreover, the administration of isomangiferin prevented the development of capillary‐like structures and the vascular endothelial growth factor (VEGF)‐induced proliferation of human umbilical vein endothelial cells (HUVECs). Isomangiferin caused breast cancer cells to undergo apoptosis in a caspase‐dependent manner. Additionally, isomangiferin inhibited the activation of the vascular endothelial growth factor receptor 2 (VEGFR‐2) kinase pathway generated by VEGF (Alkholifi et al., 2023). Research indicated that isomangiferin inhibited VEGFR‐2 functionally to prevent breast cancer. The isomanangiferin medication targets VEGFR‐2 and may be useful in treating breast cancer. An analysis of mangiferin's cytotoxic properties was conducted utilizing a human breast cancer cell culture. The fact that three different enzyme systems—the proteasome, plasmin, and 3‐hydroxy‐3‐methyl‐glutaryl–coenzyme A (HMG–CoA) reductase—were suppressed and in charge of maintaining cholesterol homeostasis, protein turnover, and cell adhesion, respectively, offered proof that mangiferin activity was multitargeting. Across the board, mangiferin was able to disrupt plasmin‐mediated methods of cell migration and induce apoptosis, which together stopped cell proliferation by targeting both the cholesterol and proteasome pathways (Cuccioloni et al., 2016).

## 5 COLON CANCER

Colon cancer is more common than rectal cancer: rates are generally comparable in nonindustrialized nations, but in industrialized nations, the ratio of colon to rectal cases is 2:1 or higher (and higher in women). Approximately 9% of all cancers in Europe are colon cancers, with over 250,000 new cases identified annually (Mattiuzzi et al., 2019). Urbanization and industrialization are associated with higher rates of colon cancer. Though it was formerly far more prevalent in high‐income nations, its prevalence is currently rising in middle‐class and low‐income nations. In most of Asia and Africa, it is still rather uncommon. Comparing Western and Northern Europe to Southern and Eastern Europe, the prevalence is marginally greater in the former. Europe, Australia, and North America are other high‐risk regions. Africa, Asia, and Central and South America are low‐risk regions (Ahmed, 2020).

The role of mangiferin in the colon carcinogenesis of albino rats induced by the chemical carcinogen azoxymethane (AOM) was investigated in a research study. The tests were carried out by the researchers: a short‐term assay to investigate the effects of mangiferin on aberrant crypt foci (ACF), which are precursors to preneoplastic lesions, and a subsequent long‐term assay to investigate the effects of mangiferin on carcinogenesis caused by AOM. Rats administered with AOM alone did not exhibit considerable reserve of ACF formation, whereas rats treated with 0.1% mangiferin in their diet did. The group that received 0.1% mangiferin treatment during the initiation phase of the experimental protocol demonstrated a significant decrease in both the multiplicity and incidence of intestinal neoplasms caused by AOM. This group's reductions were 47.3% and 41.8%, respectively, equated to the group that received AOM‐alone treatment. Rats given mangiferin had a 65%–85% decrease in colonic mucosal cell proliferation. Results of the study validated that mangiferin may have use as a naturally occurring chemopreventive substance (Rajendran et al., 2015).

By docking with many target proteins linked to the development of the illness, the molecular mechanism of action of mangiferin against colorectal cancer (CRC) was assessed. Findings from docking investigations showed good binding scores between (−6.7 and −10.1) (kcal/mol). Mangiferin had a strong attraction for enzymes involved in the metabolism of arachidonic acid (AA), such as cyclooxygenase‐2 (COX‐2) and LA4H. After mangiferin's pharmacophore feature was evaluated for COX‐2 inhibitor medications, it was further established that mangiferin shares the same pharmacophore characteristic as COX‐2 inhibitor medications. Mangiferin's effectiveness as an inhibitor, its binding affinity was likened to those of five other COX‐2 inhibitor medications. In addition, mangiferin exhibited cytotoxic activity against cell lines representing cervical cancer, breast cancer, and colorectal cancer (Samadarsi et al., 2022).

A research study was conducted to explore how colon cancer cell lines responded to mango peel extract (MPE). MPE had an impact on cell viability and reduced the tendency of tumor cells to form colonies. These events resulted from the activation of oxidative response players like JNK and extracellular signal‐regulated kinases 1/2 (ERK1/2), the induction of apoptosis linked to the formation of reactive oxygen species (ROS), and the rise in nuclear factor erythroid 2‐related factor 2 (Nrf2) and manganese superoxide dismutase (MnSOD). Mango peel‐induced stress was shown to be significant because it elicited a reaction to DNA damage, which was verified by the upregulated p53, phosphorylated ataxia telangiectasia‐mutated (ATM) kinase, and early phosphorylation of gamma histone 2AX (γH2AX).

Mango peel extract was the subject of the study, and the findings of HPLC/MS (high performance liquid chromatography/mass spectrometry) analysis indicated the existence of certain phenolic compounds that may be protected against cancer. The significance of γH2AX‐mediated genotoxic stress signaling pathway in inducing cell death in colon carcinomas is underscored by all the findings. The anticancer action of the peel extract from *Mangifera indica* L. is associated with the γH2AX‐mediated apoptosis of colon cancer cells. Researchers attempted to realize the molecular insights of mangiferin in inflammatory bowel disease (IBD) and explore the potential of mangiferin as an in vivo therapeutic in an animal model of colitis. Five percent dextran sodium sulfate (DSS) was administered for 11 days to develop colitis, and then there was a 3‐day DSS‐free interval. Colon tissues were removed for histological and biochemical examination on day 14, when the animals were killed. After day 5, when colitis was first induced, therapeutic treatment with mangiferin reduced the levels of superoxide dismutase (SOD), reduced glutathione (GSH), myeloperoxidase (MPO), and malondialdehyde (MDA), and alleviated colitis symptoms such as (weight loss and diarrhea). Histopathological score, matrix metalloproteinase‐9 (MMP‐9) activity, TNF‐α, interleukin 1 beta (IL‐1β), and other colonic pro‐inflammatory mediators were also reduced by it. GLIDE software was used to assess mangiferin's molecular docking against MMP‐9 and TNF‐α. Mangiferin's binding capability with MMP‐9 and TNF‐α was revealed by its glide scores of −9.97 kcal/mol for MMP‐9 and 8.04 kcal/mol for TNF‐α. As a result, mangiferin can provide therapeutic benefits when used to treat inflammatory bowel disease. Mangiferin decreased intestinal impairment in a mouse model with colitis and partially reduced the inflammatory and oxidative processes by directly inducing MMP‐9 and TNF‐α activities. Using a molecular docking and in vivo method, mangiferin reduces DSS colitis in mice. The downregulation of NF‐κB mediated by mangiferin showed the potential for chemotherapeutic agents to cause cell death, suggesting a potential role for combination therapy for cancer. The research study looked into the joint mechanism of mangiferin and oxaliplatin's anticancer effects. HeLa, HT29, and MCF‐7 cancer cell lines were subjected to DNA cell‐cycle studies, 3‐(2,5‐diphenyltetrazolium bromide)‐2,5‐(4,5‐dimethylthiazol‐2‐yl) (MTT) dose–response curves, trypan blue staining, and caspase‐3 assays, both with and without 10 μg/mL mangiferin added. The study exclusively used HT29 cells for NF‐κB assays, resistance induction investigations, DNA fragmentation, and mitochondrial membrane potential. In the MTT test, the addition of mangiferin (10 μg/mL) decreased the HeLa (1.7‐fold) and HT29 (3.4‐fold) cells’ oxaliplatin IC50 values while decreasing trypan blue staining. A delay in the cell‐cycle's S‐phase and an increase in caspase‐3 activation and DNA fragmentation followed this. The combo treatment did not improve the permeabilization of the mitochondrial membrane. Mangiferin has been shown to facilitate a reduction in NF‐κB activation in HT29 cells that are resistant to oxaliplatin (Lauricella et al., 2019).

## 6 LIVER CANCER

Cancer that originates in the liver is known as liver cancer. It is an aggressive tumor that often develops alongside cirrhosis and chronic liver disease. The third most common cause of cancer‐related mortality worldwide is hepatocellular carcinoma (HCC), also known as primary liver cancer. It ranks seventh among cancers in women and fifth among cancers in men. Incidences of liver cancer are rising in the US; in 2005, there were 4.5 cases per 100,000 people. Liver cancer is still one of the most challenging malignancies to treat, even with advancements in medical care. Surgery, local destructive therapy, and liver transplantation offer viable cures for people with early‐stage HCC. Even with curative treatment, HCC recurrence is still a significant issue—it reaches a frequency of over 70% after 5 years. Surgically treated patients with early‐stage, tiny HCC (<3 cm) have an unsatisfactory 5‐year survival rate (47%–53%) (Dhar et al., 2020). The majority of individuals with advanced stage HCC are not eligible for curative therapy, and the disease is typically identified at an advanced stage. Furthermore, there are minimal survival advantages and poor effectiveness rates with conventional systemic chemotherapy. The approval of sorafenib, a multikinase inhibitor, demonstrated some benefit to survival in patients with advanced HCC who have retained liver function, indicated that molecular targeting of advanced HCC is a promising treatment option therapy for hepatic cancer. Liver cancer ranks as the sixth most common cancer worldwide, with a 9.1% annual death rate. The prevalent kind of primary liver cancer, hepatocellular carcinoma, primarily affects those with cirrhosis and chronic liver disease. Since cancer is typically discovered only after it is advanced, treatments like radiation, ablation, and chemotherapy are frequently used to treat it, which has a poor prognosis and causes severe side effects (Anwanwan et al., 2020).

Mangiferin's anticarcinogenic properties against diethylnitrosamine (DEN)‐induced hepatocellular cancer were examined in the research investigation. Healthy Sprague–Dawley rats were given 0.01% diethylnitrosamine (DEN) in their drinking water for a period of 12 weeks. The rats were then administered 50 mg of mangiferin for an additional 8 weeks, with the aim of causing hepatocellular cancer. The liver of DEN^−^ and DEN^+^ mangiferin‐treated rats was examined for biochemistry, oxidative stress markers, antioxidant status, and tumor marker level in order to assess the efficacy of mangiferin in preventing the development of cancer.

**FIGURE 3:**
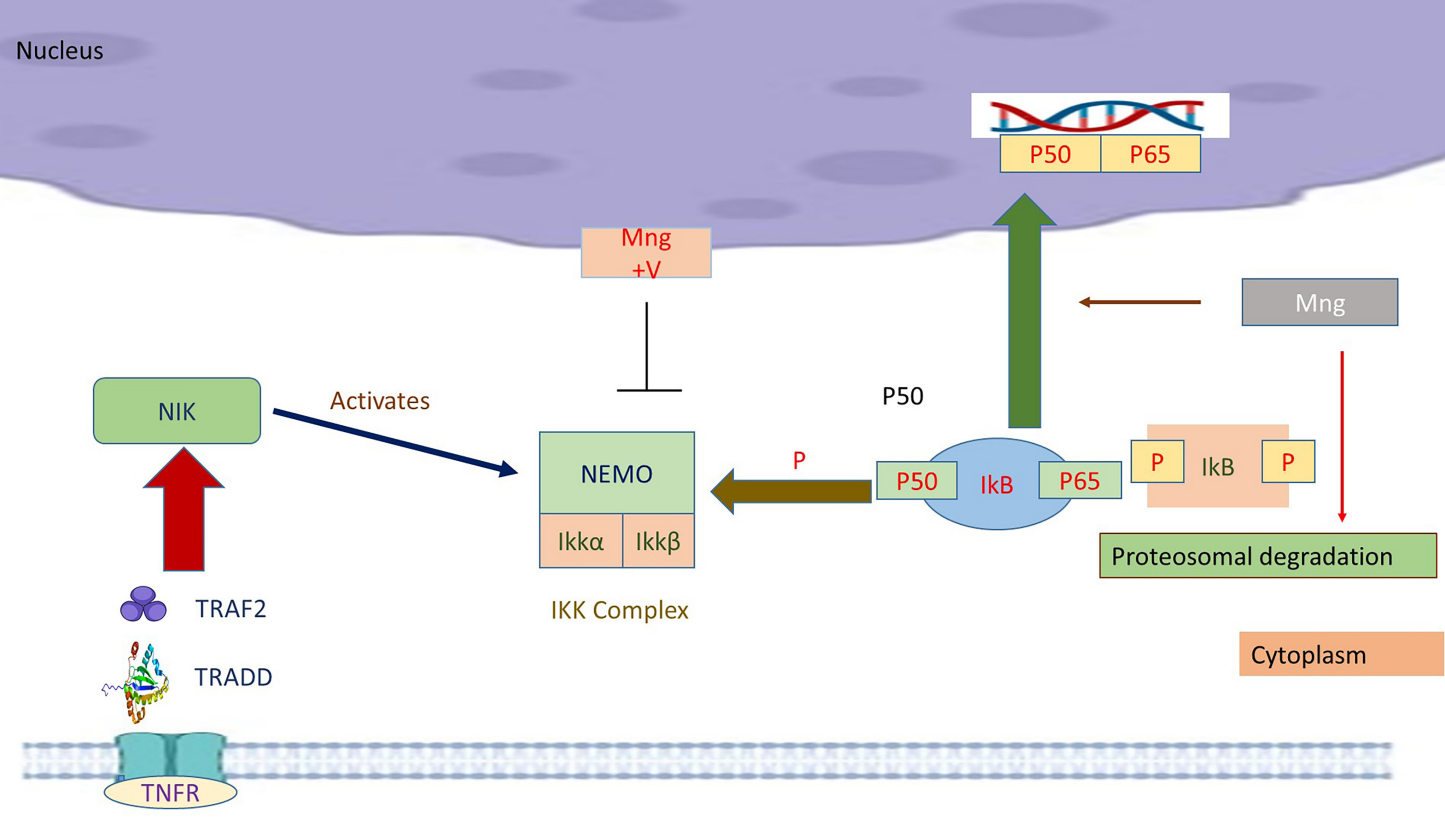
Mangiferin inhibits cancer cells’ proliferation by multiple signaling pathways and cellular processes.

By evaluating the appearance of apoptotic proteins and performing a histological examination on liver tissue from rats treated with DEN and DEN^+^ mangiferin, the anticarcinogenic potential of mangiferin was authenticated. The anticarcinogenic activities of mangiferin against DEN‐induced hepatocellular carcinoma were demonstrated by the results. In Sprague–Dawley rats, mangiferin reduced diethynitrosamine‐induced hepatocellular cancer by changing the oxidative stress and apoptotic pathways. Hepatocellular carcinoma formation is a multifaceted process involving the dysregulation of several signaling pathways. The persistent growth, invasion, and neovascularization of HCC are encouraged by the hyperactivation of Wnt signaling. Mangiferin has demonstrated the ability to deactivate β‐catenin, an essential regulator in the Wnt pathway (Yang et al., 2019). Figure 3 shows that the multiple signaling pathways and cellular processes of mangiferin inhibit cancer cells’ proliferation.

An interesting research study looked into how mangiferin affected Wnt signaling and whether it had any inhibitory effects on HCC. The tumor‐inhibitory effect of mangiferin was examined using both in vitro cellular models and an in vivo orthotopic HCC implantation model. Polymerase chain reaction (PCR) microarray technique was engaged to classify the genes responsible for mangiferin‐mediated anti‐HCC. In order to evaluate the target genes’ expression further, PCR and immunoblotting techniques were employed. The capacity of Wilms’ tumor 1 (WT1) to bind to the lymphoid enhancer‐binding factor 1 (LEF1) promoter was demonstrated by chromatin immunoprecipitation–quantitative polymerase chain reaction (qPCR). Oral mangiferin treatment prevented the formation of orthotopic tumors. Cellular investigations confirmed that mangiferin inhibits HCC invasion and growth in a dose‐dependent manner. Mangiferin primarily targets the Wnt pathway, with LEF1 being the most decreased gene in the Wnt pathway, according to PCR array and Gene Ontology (GO) analysis. When HCC cells were treated with mangiferin, overexpression of LEF1 decreased Wnt signaling suppression and decreased proliferative activity. The downregulation of LEF1 induced by mangiferin was linked to WT1 protein but not dependent on β‐catenin. In HCC cells, WT1 knock‐in increased LEF1 expression even more. The results revealed a new way of inhibiting hepatocellular cancer through Wnt signaling that was independent of β‐catenin and controlled by WT1‐associated LEF1 suppression. Mangiferin was another Wnt inhibitor that the study suggested a good option for treating HCC. In hepatocellular cancer, WT1‐mediated LEF1 transcription is repressed by mangiferin, which controls β‐catenin‐independent Wnt signaling inactivation (Husna et al., 2022).

Researchers examined the molecular mechanisms underlying mangiferin's protective effect in contradiction of lead‐related hepatic pathology. Lead nitrate (Pb(NO_3_)_2_) is the form of lead [Pb(II)] administered orally for 6 days five milligrams per kilogram of body weight resulted in cell death, hepatic dysfunction, and oxidative stress in the mice liver. Instead, reactive oxygen species production was decreased and serum marker enzyme levels of alkaline phosphatase (ALP) and alanine aminotransferase (ALT) were decreased after oral mangiferin administration during 6 days, at a dose of 100 mg/kg body weight. Mangiferin reversed the effects of Pb(II) on antioxidant mechanisms, B‐cell lymphoma‐2/Bcl‐2‐associated X protein (Bcl‐2/Bax) mutual regulation and mitochondrial membrane potential. Likewise, mangiferin reserved Pb(II)‐induced initiation of nuclear translocation of NF‐κB, mitogen‐activated protein kinases (MAPKs) (phospho‐ERK 1/2, phospho‐JNK, and phospho‐p38), and apoptotic cell death, as shown by fluorescence‐activated cell sorting (FACS) analysis, DNA disintegration, and histological examination. Hepatocytes were used as the working model in in vitro tests, which further demonstrated that mangiferin guards against Pb(II)‐induced cytotoxicity. The collective beneficial effects of mangiferin (II) have led to a notable reduction in Pb‐induced apoptotic liver cell death. Overall, the results demonstrated that mangiferin exhibited both anti‐apoptotic and antioxidative properties, protecting the body part from hepatic impairment induced by Pb(II). MAPK, NF‐κB, and mitochondria‐dependent pathways are used by a naturally occurring xanthone named mangiferin to protect the liver of mice against Pb (II)‐induced hepatic damage and cell death (Tao et al., 2023).

## 7 GASTRIC CANCER

Gastric cancer is the sixth most common cancer in the world to receive a diagnosis, with more than a million fresh cases recorded annually. With 784,000 deaths worldwide in 2018, stomach cancer ranks third among cancer‐related deaths due to its high mortality rate, which is often associated with advanced stages at diagnosis. There are regions in South America, Eastern Europe, and East Asia where stomach cancer is most common and fatal. The risk of gastric cancer is twofold as high in both men and women (Smyth et al., 2020). The death and incidence rates of this tumor have consistently reduced throughout the last century. Because of population aging, doctors should anticipate seeing an increase in stomach cancer cases in the future, even if incidence rates are dropping in the majority of countries. The main cause of gastric cancer, *Helicobacter pylori*, has become less common as a result of improved living conditions brought about by economic growth. Screening initiatives have also significantly decreased the mortality rate associated with stomach cancer in high‐incidence regions like Korea and Japan. A shift in the disease risk and epidemiology of stomach cancer is suggested by an increase in incidence that has been seen in younger individuals from high‐income nations. Therefore, it is vital to future cancer control and therapeutic practice to pay attention to continuous changes in the epidemiology of stomach cancer (Thrift & El‐Serag, 2020).

To assess mangiferin's antiproliferative activity, the MTT test was used. After treatment, western blot analysis was utilized to identify the appearance of proteins connected to programmed cell death, while laser scanning confocal microscopy (LSCM) and flow cytometry were hired to ascertain the apoptosis rates of SGC‐7901. Mangiferin inhibited SGC‐7901 and BCG‐823 cell proliferation in a time‐ and dose‐dependent manner, as the MTT assay showed. For SGC‐7901 cells, the values of the half‐maximal inhibitory concentration (IC50) of mangiferin were 4.79, 8.63, and 16.00 μmol/L after 24, 48, and 72 h, respectively. Mangiferin‐induced apoptosis in SGC‐7901 cells was detected using positive staining for terminal deoxynucleotidyl transferase dUTP nick end labeling (TUNEL) and Annexin V/propidium iodide (PI) double staining. The expression of Bax, Bcl‐2‐associated death promoter (Bad), cleaved caspase‐3, ‐9, and Bcl‐2 expression was evidently elevated, whereas B‐cell lymphoma‐extra large (Bcl‐xL) and induced myeloid leukemia cell differentiation protein (Mcl‐1) significantly decreased after mangiferin treatment of SGC‐7901 cells. Additionally, mangiferin expressively reduced the levels of phospho‐mammalian target of rapamycin (p‐mTOR), phospho‐protein kinase B (p‐Akt), and phospho‐phosphatidylinositol 3 kinase (p‐PI3K) in SGC‐7901 cells treated with *epidermal* growth factor (EGF), but had no effect on PI3K, Akt, or mTOR. It is noteworthy that LY294002 significantly worsened the apoptotic and growth‐inhibitory effects of mangiferin, whereas the Akt activator SC79 somewhat mitigated the mangiferin's proapoptotic action on SGC‐7901 cells. Summarized, the study demonstrated that mangiferin effectively constrains the growth of cancer cells that are gastric and encourages them to undergo programmed cell death over the inhibition of the PI3K/Akt pathways. This implied that a new chemotherapeutic medication called mangiferin may be used to treat stomach cancer (Du et al., 2018).

**FIGURE 4:**
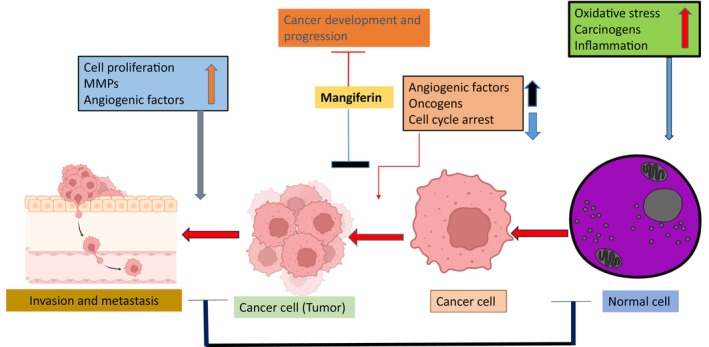
Anticancer cellular pathways modulated by mangiferin.

The objective of the investigation was to confirm the anti‐*H. pylori* effectiveness of mangiferin on AGS cells diseased with *H. pylori*. To evaluate the anti‐*H. pylori*'s impact of either amoxicillin (AMX) and dimethyl sulfoxide (DMSO) (control) group or mangiferin (10, 20, 50, and 100 μg/mL) on co‐cultured human gastric adenocarcinoma cell line (AGS) cells and *H. pylori*, several inflammatory markers were examined, along with the inhibitory zone, bacterial drug sensitivity test (Minimum Bactericidal Concentration (MBC) and Minimum Inhibitory Concentration (MIC)), invasive and adhesion property, and various inflammatory markers. Mangiferin (100 μg) was added on to *H. pylori*‐infected AGS cells during co‐culturing, and this meaningfully expanded the inhibitory zone, significantly decreasing MBC and MIC levels, and condensed adhesion and invasiveness in a way that is dependent on dosage, supporting the medication's anti‐*H. pylori* activity. Mangiferin administration also resulted in a significant suppression of inflammatory markers, including TNF‐α, IL‐8, NF‐κB subunit p65, and interleukin‐1β as shown in Figure 4 (Kaurav et al., 2023).

Researchers revealed that administering mangiferin to AGS cells infected with *H. pylori* significantly delayed the adhesion and invasion processes. It inhibited the inflammatory response and lowered the risk of gastric cancer by deactivating NF‐κB p65. The effect of mangiferin on irritation was created by *Helicobacter pylori* in human gastric cancer AGS cells. Mangiferin was tested in mice on stomach damage brought on by ethanol and indomethacin. Measures of stomach secretory volume and total acidity in 4‐h pylorus‐ligated rats, as well as changes in mean gastric lesion area or ulcer score in mice, were employed to assess the impact of mangiferin on gastric mucosal damage. Mangiferin (3, 10, and 30% mg/kg) significantly reduced the amount of stomach damage induced by ethanol (3%, 10%, and 30%) and indomethacin (22%, 23%, and 57%), respectively. N‐acetylcysteine (750 mg/kg) and lansoprazole (30 mg/kg), the positive controls in these ulcerogenic mice, resulted in 50% and 76% suppression of stomach injury, respectively. In 4‐h pylorus‐ligated rats, mangiferin (30 mg/kg) injected intramedullary led to significant decreases in the overall acidity and stomach secretory volume. Furthermore, mangiferin dramatically decreased the reduction of nonprotein sulfhydryl concentration in the stomach mucosa in mice that was caused by ethanol, suggesting that it has an antioxidant effect akin to that of N‐acetylcysteine (NAC), a source of sulfhydryls. Results validated that mangiferin provided gastroprotection against stomach damage brought on by ethanol and indomethacin, perhaps through its antisecretory and antioxidant modes of action (Chi et al., 2021).

## 8 OVARIAN CANCER

The most aggressive gynecologic malignancy is ovarian cancer and has the worst 5‐year survival rates among all types of the cancer in women. The potential source of one of the ovary's three main components—the stroma, germinal cells, and epithelium—determines the classification of ovarian cancer. Although ovarian cancer has not increased in frequency globally in recent decades, it is nevertheless a disease that has been linked to a significant number of deaths worldwide (Lee et al., 2019).The epidemiology of this cancer demonstrates racial and national disparities because of a number of factors, including economic and genetic. The global numbers for incidence and mortality vary depending on the location under study, coming in at 6.6 and 3.9 per 100,000, respectively. It has been difficult to diagnose this cancer since there is no public screening program for early diagnosis. The majority of ovarian malignancies are therefore found at an advanced stage, frequently after they have migrated to other parts of the body. Mangiferin hinders human ovarian cancer cells through its interaction with Notch3, which has an antineoplastic impact. Additionally, yes‐associated protein (YAP) has been demonstrated to be a functionally relevant downstream effector of Notch signaling; hence, a hypothesis was made that YAP may be related to the anticancer impact of mangiferin (Momenimovahed et al., 2019).

The intention of the research was to investigate the molecular mechanism of mangiferin's anticancer activity and to explain the inhibitory consequence of mangiferin on ovarian melanoma. Reflecting the in vitro analysis and markedly diminished cell proliferation of the mangiferin‐exposed cells relative to the cells overexpressing YAP, subjected to mangiferin treatment, it was determined that mangiferin significantly downregulates YAP expression. On the other hand, it was found that YAP overexpression may negate the antitumor efficacy of mangiferin based on measures of cell morphology and apoptotic percentages. Furthermore, cells treated with mangiferin had decreased invasion and migration, both of which can be avoided by overexpressing YAP. These results therefore offered additional proof that mangiferin hampers metastatic dissemination by governing YAP. Researchers assessed the OVCAR8 cells’ resistance to cisplatin and discovered that mangiferin may augment the drug susceptibility of tumor cells; YAP overexpression also eliminated this enhanced sensitivity. All of these findings suggested that YAP not only facilitated drug resistance in tumor cells but was also strongly linked to the anticancer impact of mangiferin. Likewise, the mangiferin‐treated cells showed a diminishment of downstream TEA domain transcription factor 4 levels, validating the obstructive impact of mangiferin on YAP. Furthermore, OVCAR8 cell xenograft models illustrated that mangiferin repressed tumor development and prolonged the lifetime of tumor xenograft mice by making a tumor more sensitive to cisplatin. As a tumor xenograft model system, athymic mice were taken to examine the effect of YAP on the antineoplastic attributes of mangiferin and the heightened tumor susceptibility to cisplatin triggered by mangiferin. After 20 days of therapy, the xenograft neoplasm size was substantially less in the mangiferin group, and the empty vector combination than among the group that received treatment with cisplatin alone. Conversely, in the set receiving cisplatin with mangiferin treatment, the size of the xenograft tumor increased and YAP was overexpressed. The xenograft tumor weights of the mice administered with empty vector, cisplatin, and mangiferin values were substantially lower than those of the cisplatin monotherapy group, which indicates that mangiferin has the potential to increase sensitivity of some type of ovarian tumors to cisplatin. Comparing the mice within the cisplatin monotherapy group with those receiving mangiferin plus cisplatin, they had a substantial upsurge in body mass, which was in line with the reduced tumor size. Furthermore, the mice treated with cisplatin and YAP‐overexpressed mangiferin survived after 15, 17, 26, 53, and 69 days. The mangiferin and cisplatin‐treated mice had survival durations of 36, 39, 75, 100, and 100 days. On the other hand, mice in the control group that were given only cisplatin have perished durations of 1, 15, 33, 53, 79, and 100 days. Based on the ability to endure results, mice treated with ovarian cell xenografts may live longer than mice in any other group if mangiferin is given, in addition to cisplatin treatment. In vivo findings revealed that sensitivity of cisplatin was augmented by mangiferin by blocking the YAP route (Kuroki & Guntupalli, 2020).

Ultimately, the aim of the research was intended to find out in what way mangiferin inhibited human ovarian cancer epithelial cell and elucidated the underpinning chemical pathways. To ascertain the half maximal values for the inhibitory concentration (IC50) of mangiferin, the MTT assay, which uses 3,(4,5‐dimethylthiazol‐2‐yl)‐2,5‐diphenyltetrazolium bromide (MTT), was used with paclitaxel acting as a positive control. Mangiferin inhibited the development of two human epithelial ovarian cancerous cell lines. To assess the antimetastastic characteristics of mangiferin transwell assays, wound healing was employed. For the in vivo research, models of Nude ES‐2 xenograft mice were put to utilize. Immunohistochemistry (IHC) was adopted for estimating the expression levels of matrix metalloproteinase 2 (MMP2) and matrix metalloproteinase 9 (MMP9), the Western blotting approaches and the enzyme‐linked immunosorbent assay (ELISA) test. In both vivo and in vitro studies, mangaferin inhibited the proliferation of epithelial ovarian cancer cells. In the mangiferin‐treated rats, the weight and volume of the tumors significantly decreased. Mangiferin diminished the proliferation of the proteins MMP2 and MMP9, which are interconnected to metastasis, in accordance with analyses of the relevant molecular events. Mangiferin reduced the expression of MMP2 and MMP9, which considerably slowed the development of human epithelial ovarian cancer (Zeng et al., 2020).

## 9 PROSTATE CANCER

One very common cancer that affects the male reproductive system is prostate cancer. After lung cancer, it illustrates 358,989 deaths (3.8% of all male cancer‐related deaths) and 1,276,106 new cases of the second prevalent cancer in men across borders. The average age of diagnosis for prostate tumor is 66, and death rates from the disease rise with age worldwide (Rawla, 2019). Compared to White males, a combination of 158.3 new cases acknowledged per 100,000 men, African‐American men had higher prevalence rates—and practically twice as high deceased rate. Early prostate cancer often progresses slowly with little or no symptoms and requires little or no treatment. Prostate tissue typically yields the glycoprotein, referred to as prostate‐specific antigen, or PSA. It is found in high concentrations in plasma and is used to diagnose various types of prostate cancer. It has been shown that men without cancer also have elevated PSA results, hence the gold standard of care for determining the presence of cancer is a tissue biopsy (Litwin & Tan, 2017).

It was discovered that mangiferin had the ability to impede PC3‐cell proliferation in accordance with a methodology that was reliant on both concentration and time. Researchers found that mangiferin can cause early apoptosis and G2/M arrest, which would prevent the growth of human nasopharyngeal cancer cells. Human PC3 prostate cancer cells, mangiferin, in an approach that entails attention, increased the caspase‐3 activity and accelerated the apoptotic rate. When combined, these findings suggest that in cancer cell lines, mangiferin may stimulate caspase‐3 activation and induce apoptosis. Important genes involved in the control of apoptosis are found in the family of Bcl‐2 proteins. By managing the functioning of mitochondria, the foremost BCL‐2 family members, that are pro‐ and anti‐apoptotic, establish apoptosis. By stopping the concentration of cytoplasmic calcium from rising, Bcl‐2 suppresses apoptosis. It is currently believed that Bcl‐2 is an anti‐apoptotic gene that, by delaying apoptosis and prolonging cell life, promotes the development of malignancies. Previous studies have demonstrated that Bcl‐2 evincement is a feature that is significant in certain human malignant tumors. In PC3 cells, mangiferin effectively lowered the levels of Bcl‐2 expression. Researchers found that the production of Bcl‐2 and the X protein linked with Bcl‐2 (Bax) was the mechanism by which mangiferin promoted cell death. Mangiferin inhibits Bax and Bcl‐2 expression to reduce diabetic nephropathy. The guideline of gene expression, which is crucial for cell growth, differentiation, apoptosis, and cancer, is facilitated by a group of RNA small molecules known as microRNAs (miRs). The genomic region of a tumor‐associated or fragile premises incorporates more than half of the human miR genes. The exposition of particular miRs could possibly be a good indicator of the extent of differentiation and the stage of the tumor, according to research using expression profiling. In PC3 cells used in this investigation, mangiferin administration dramatically raised the levels of microRNA‐182 (miR‐182) expression. Notably, the expression of Bcl‐2 was decreased in PC3 compartments by upregulating miR‐182. Also, PC3 cells’ expression of Bcl‐2 increased and mangiferin's impact was lessened when miR‐182 expression was suppressed. Following atorvastatin therapy, PC3 human prostate cancer cells’ ability to proliferate is inhibited, as a result of downregulating Bcl‐2 and upregulating miR‐182. It has been noted that overexpression of miR‐182 suppresses the expression of Bcl‐2, thereby hampering the advancement of uveal melanoma cells (Akter et al., 2022).

Mangiferin dramatically eradicates the nuclear evacuation of the NF‐κB subunits, p65 and p50, diminishes NF‐κB activity, and reduces TNF‐α‐induced MMP‐9 activity in androgen‐sensitive individuals with LNCaP (lymph node carcinoma of the prostate) cancer cells. It is also commonly recognized that MMP‐7 and MMP‐9 dramatically quicken the course of cancerous tumor cell growth and development. Nevertheless, in PC3 prostate cancer cells, mangiferin substantially boosts the expression of miR‐182, while enormously eliminating Bcl‐2‐expression, and stimulates caspase‐3 activity and apoptosis (Khoobchandani et al., 2021).

In the research study, antiproliferative activity was assessed using the MTT test in order to look into possible impacts of mangiferin on PC3 at 0, 10, 20, and 40 μM cell growth. In a concentration‐dependent way, mangiferin slowed PC3 cell growth. The PC3 cells’ rate of proliferation was considerably lower when they were treated with 20 μM mangiferin for 72 h, or with 40 μm mangiferin for 48 or 72 h, in comparison toward the group under control that received 0 μM mangiferin. Apoptosis plus caspase‐3 activity was slow in order to assess the impact of mangiferin (0, 10, 20, and 40 μM) on PC3 cell apoptosis. Mangiferin boosted PC3 cell caspase‐3 activity and expedited apoptosis in accordance with concentration. Besides, after subjecting PC3 cells to mangiferin (20 and 40 μM) for 72 h, there was a notable rise in apoptosis as compared to the group pursuant to control, resulting in acquired 0 μM mangiferin treatment. When PC3 cells treated with 20 and 40 μM mangiferin appeared compared to the control group treated with 0 μM mangiferin, the caspase‐3 activity of the initial group was tremendously enhanced (Hans et al., 2022).

The expression of Bcl‐2 was examined to clarify the impact of mangiferin on Bcl‐2 expression and PC3 cells were treated to 0, 10, 20, and 40 μM mangiferin for 72 h. After being unprotected to mangiferin at all doses, it was suppressing Bcl‐2 protein expression. When compared to the control group's cells treated with 0 μM mangiferin, the detected levels of Bcl‐2 expression in PC3 cells seemed significantly decreased by it at 20 and 40 μM (Sarfraz et al., 2023).

## 10 BLOOD CANCER

Leukemia is the collective terminology for malignancies of the plasma cells. The blood cell type that transforms cancerous and the rate of spread identify the particular type of leukemia. While leukemia does strike adults over the age of 55 more commonly, it primarily affects youngsters under the age of 15. With 35,000 new cases reported each year, leukemia is the tenth most prevalent disease in the United States (U.S.) (Nor Hisam et al., 2021). The most common theory regarding leukemia is that it arises from changes (mutations) that some blood cells experience in their DNA or genetic composition. Over time, leukemia signs and symptoms are triggered by the surplus of erythrocytes and leukocytes and platelets, which are produced when healthy blood cells are pushed out of the bone marrow by abnormal blood cells. People who had undertaken certain types of treatment for other malignancies, radiation, and chemotherapy are more prone to attain certain types of leukemia (Bukhari et al., 2022).

Mangiferin showed antagonist of leukemia and preventative impacts in HL‐60 leukemia cells. Mangiferin promoted the cellular cycle arrest during the G2/M phase by dose‐dependently regulating the signaling route between cyclin B1 and CDK1 (cyclin‐dependent kinase 1). Elevated dosages caused Wee1 mRNA expression to be upregulated, cdc25C and Chk1 (checkpoint kinase 1) mRNA expression to be significantly suppressed, and ataxia telangiectasia and Rad3‐related protein (ATR), Erk1/2, Wee1, Chk1, and Akt are hyperactive to be significantly inhibited. Additionally, mangiferin inhibited the adrenaline rush of CDC25C and cyclin B1, as well as levels of protein production for Wee1 and Akt. This was accomplished by ATRChk1. The drug magniferin is reliant on both dose and time that boosts the protein and expression of Nrf2 (nuclear factor erythroid 2‐related factor 2) stability in HL‐60 human myeloid leukocytes. Additionally, it made the Nrf2 protein more stable, which stopped blood cells from multiplying and degrading (Shang et al., 2021).

Mangiferin (50 μM) within HL‐60 cells increases the buildup of Nrf2 protein, improves Nrf2 affixing itself, is intended to AREs (antioxidant response elements), modifies the production of NAD(P)H:quinone oxidoreductases, or NQO1, and limits the ranges of intracellular ROS. Additionally, it reduced oxidative stress and alleviated the cytotoxicity caused by etoposide within human umbilical cord blood mononuclear cells (HUCB‐MNCs). Mangiferin's capacity to activate the Nrf2–ARE pathway contributes significantly to its capacity to minimize DNA damage. Chemotherapeutic drugs can be prevented from acting on normal cells by the Nrf2–ARE pathway. Nevertheless, cancer cells that overexpress Nrf2 may become more resistant to treatment. HL‐60 cells with acute myeloid leukemia, KY821 cells, and KG‐1 cells all showed decreased viability when exposed to mangiferin (100 μg/mL). Both caspase‐3 activity and DNA fragmentation increased concurrently. Other survival signals’ demonstration including protein kinase B (Akt), additionally referred to as extracellular signal‐regulated kinase 1/2 (ERK 1/2), and p38 mitogen‐activated protein kinase (MAPK), remained unchanged when mangiferin was administered, despite a considerable reduction in the nuclear uptake of NF‐κB p65. X‐chromosome‐linked inhibitor of apoptosis protein (XIAP) and Bcl‐xL are inhibited by mangiferin, but not by Bcl‐2 Interacting Mediator of cell death (BIM) is additionally recognized as Bcl‐2, Bcl‐2‐associated X protein, or BCL‐2. These findings verified that mangiferin triggers apoptosis through blocking NF‐κB activation and Bcl‐xL (B‐cell lymphoma‐extra large) immunoglobulin expressions, and expressions of XIAP. Subsequently, acute myeloid leukemia can be treated with mangiferin either alone or in conjunction with other anticancer medications (Peng et al., 2015).

Mangiferin most likely prevents the manifestation of the BCR/ABL chromosomal factors within a quantum and chronological dependency across a spectrum of concentrations (25–200 μmol/L), stopping the onset of apoptosis in the K562 cell line and the maturation of K562 chronic myeloid leukemia cells. The mechanisms behind the restriction of HL‐60 cells include the augmentation that comprises a decline in cell‐cycle progression at the G2/M phase and a leap in the expression of CDC2 and Cyclin B (CCNB1) mRNA. The inquiries were executed on human HL‐60 myeloid leukemia cells and human umbilical cord blood mononuclear cells (HUCB‐MNCs). The Nrf2 protein was identified availing of Western blotting and immunoillumination labeling. By using the electrophoretic mobility shift experiment, evaluation was executed on Nrf2's binding to ARE. Real‐time RT‐PCR and Western blotting were implemented in order to evaluate NQO1 levels. With the aid of 2′−7′‐ dichlorodihydrofluorescein diacetate (DCFH‐DA), the intracellular ROS level was determined. To evaluate apoptosis and cell proliferation, subsequently, flow cytometry and MTT were implemented. The HL‐60 cells’ buildup of Nrf2 protein was considerably enhanced by mangiferin (50 μmol/L), especially in the nucleus. Mangiferin additionally decreased intracellular ROS in HL‐60 cells, markedly increased NQO1 expression, and improved Nrf2 binding to an ARE. HL‐60 cell growth was dose‐dependently reduced by mangiferin alone. The deleterious impacts of etoposide on HL‐60 cells were not reduced by mangiferin (50 mol/L), and the cell inhibition rate was even elevated when mangiferin and etoposide were treated together at a low dose (0.8 μg/mL). In HL‐60 cells, mangiferin had no effect on the pace of apoptosis produced by etoposide. While attenuating the cytotoxicity generated by etoposide, mangiferin dramatically reduced oxidative stress in MNCs. A new Nrf2 activator called mangiferin lowers oxidative stress and shields normal cells from HL‐60 leukemia cells’ sensitivity to etoposide in vitro (Ayatollahi et al., 2019).

Researchers inquired into precisely how mangiferin hampered leukemia. Leukemia was triggered by WEHI‐3 cells in BALB/c mice. The immunological experimentation on behaviors and survival rate comprised the two primary components of the trials. Each component consisted of 40 rats that were casually allocated to five collections (*N* = 8) in order to examine the survival rate and immune response. Groups II–V comprised leukemia‐producing WEHI‐3 cells, whereas group I comprised of typical animals. Group II mice were provided with a standard diet as a reliable control. Mice in groups III, IV, and V were administered intraperitoneal injections of mangiferin at concentrations of 40, 80, and 120 mg/kg, for a duration of 20 days. Using flow cytometry, the leukocyte, natural killer (NK) cell exertion, macrophage phagocytosis, and cell population were inquired. Concanavalin A (Con A) and lipopolysaccharide (LPS)‐treated isolated splenocytes were utilized for tracking the proliferation of B and T cells, respectively. The splenocytes were then subjected to flow cytometry analysis. The weights of the leukemia mice's liver and spleen were shown to be greatly reduced by mangiferin, but their body weight grew significantly. Additionally, mangiferin boosted the quantity of both CD19 B cells and CD3 T cells, although lowering that of macrophages Mac‐3 and monocytes CD11b. Moreover, at 40, 80, and 120 mg/kg of therapy, mangiferin reduces the macrophages from the peritoneal cavity and peripheral blood mononuclear cells (PBMCs) are devoured. NK cell activity was nevertheless also elevated at 40 and 120 mg/kg of therapy. At the three levels tested, there were no impacts on the proliferation of T and B cells. Mangiferin dramatically increased the probability of leukemia‐mice surviving, receiving 40 and 120 mg/kg of treatment in vivo, according to research on survival rates (Morozkina et al., 2021).

## 11 LUNG CANCER

Lung adenocarcinoma (LUAD) accounts for approximately 45% of instances of lung cancer (LC), a highly diverse disease that causes roughly a quarter of fatalities from cancer worldwide Due to absence of methods along with distinguishable symptoms for early identification, over 60% of lung cancer patients receive their diagnosis at an advanced or locally metastatic stage, and for these patients, standard surgery may not be a beneficial alternative. Despite significant advancements in treatment approaches improving the outlook for certain LUAD patients, less than 20% of patients survive for 5 years on average. Lung cancer cases increased to around 2.2 million in 2020. Lung and bronchus cancer patients in the US were predicted to number 603,989 as of 2020. Eighty to ninety percent of cases of lung cancer are related to tobacco usage. Living a lifetime of smoking increases one's risk of lung cancer by 20–30 times compared to never smoking (Thandra et al., 2021).

The intention behind the research was done to elucidate the in vitro mechanism on mangiferin and its biological impact on LUAD cells. It has been demonstrated that A549, H1299, and H2030 cell lines, which are representative of lung adenocarcinoma (LUAD), exhibit a significant reduction in growth in a time‐ and dose‐dependent manner following exposure to mangiferin. Mangiferin also possessed the capacity to cause apoptosis; in fact, a higher percentage of cells were stopped within the G1‐ and S‐phases when exposed to mangiferin than when not. LUAD tissues exhibited increased levels of miR‐27b and miR‐92a compared to non‐LUAD tissues, as determined by microarray and microRNA sequencing analyses. Subsequent research revealed it to be possible that mangiferin is connected to the miR‐92a and miR‐27b downregulated levels. Finally, mangiferin was most expected to adjust the growth as well as cause apoptosis of LUAD cells manufactured by downregulating the miR‐92a and miR‐27b expression (Zhou et al., 2022).

Mangiferin's effects were examined in both the pre‐ and post‐initiation stages using mice that bore lung adenocarcinoma. Throughout the investigation, male Swiss albino mice in good health (6–8 weeks old) were employed. Two weeks prior to the development of benzo[a]pyrene [B (a) P]‐induced lung injury at 50 mg/kg body weight cancer and that twelfth week following it (postinitiation) were the times the mice received mangiferin treatment (dissolve 100 milligrams per kilogram of body weight in maize oil). In the animals exposed to carcinogens, body weight dropped, lung capacity increased, and the liver marker enzyme and xenobiotic levels significantly grew. After receiving mangiferin treatment, these parameters returned to values that were almost normal. Additionally, the lysosomal enzyme activities in the mice with experimental lung carcinogenesis triggered by B (a) P were evaluated. N‐acetyl glucosaminidase, β‐galactosidase, β‐glucuronidase, and acid phosphatase were among the lysosomal enzymes that showed increased activity in these animals. These changes demonstrated the anticancer properties of mangiferin (Spampinato et al., 2022).

The study's objective was to ascertain the impact about mangiferin toward apoptotic process of the human lung cancer group A549. In vitro *research* presented that mangiferin‐inhibited propagation too generated programmed cell death in A549 cells. In vivo in xenograft mice A549, mangiferin presented antitumor effects. By inhibiting the cyclin B1‐cyclin‐dependent kinase 1 signaling pathway, mangiferin produced G2/M phase cell‐cycle arrest. It caused nucleus death by obstructing the route used by nuclear factor‐κB–protein kinase C (PKC) (Naraki et al., 2021).

Mangiferin showed notable anticancer benefits in vivo, as evidenced by its capacity to prolong the survival of xenograft mice. Mangiferin may be used as an antineoplastic medication in the future to treat cancer. It elucidated the molecular processes that underlie mangiferin's antitumor effects as anticipated. The main emphasis of the research analysis was the mechanism of its anticancer activity on NCI‐H520 nonsmall‐cell lung cancer (NSCLC) cells and LPS‐induced A549 cells. In order to ascertain the growth inhibitory effect of mangiferin against LPS‐induced NSCLC cells, 3‐(2,5‐diphenyltetrazolium bromide)‐2,5‐(4,5‐dimethylthiazol‐2‐yl) (MTT) was utilized. Furthermore, mangiferin suppressed that release on IL‐1β in NSCLC cells caused by LPS and strongly mediated the protein levels on Period 1 (PER1) and nucleotide‐binding domain, leucine‐rich‐containing family, pyrin domain containing 3 (NLRP3). These findings recommend that mangiferin possesses a potent anti‐inflammatory and antitumor agent, suggesting anti‐inflammatory and anticancer medications (Ye et al., 2023).

The purpose of the study responded to develop that powerful cancer treatment that stopped oxidative stress, a major cancer cause. In Swiss albino mice, 50 mg kg^−1^ b/w oral benzo(a)pyrene was researched for its impact on lung carcinogenesis. Researchers explored the mangiferin's modulatory influence on mitochondrial lipid peroxidation (LPO), key enzymes in the electron transport chain and the tricarboxylic acid cycle (TCA) complexes. Mice with lung cancer exhibited decreased activity to major complexes of the electron transport chain and TCA cycle enzymes like malate dehydrogenase (MDH) or succinate dehydrogenase (SDH), isocitrate dehydrogenase (ICDH), and α‐KGDH stands for alpha‐ketoglutarate dehydrogenase. Using mangiferin (100 mg kg^−1^ b/w orally) before and after therapy for 18 weeks prevented the previously mentioned biochemical alterations and leaned toward normal control animal values (Razura‐Carmona et al., 2022).

## 12 KIDNEY CANCER

A prevalent urological disease that puts a heavy strain on healthcare systems, particularly in Western nations, is kidney cancer. Renal parenchyma gives rise to kidney cancer. Clear cell renal cell carcinomas in adults are responsible for around 70% on instances of renal cancer. The significant regional and temporal differences in incidence rates are the primary epidemiologic features of kidney cancer. Only body size, history of hypertension, and chronic renal disease are included in the list of recognized risk factors (Linehan et al., 2019). At 2.4% of all cancers, kidney cancer ranks 13th in the world's most prevalent cancers, with over 330,000 new cases identified each year. In Europe, North America, Australia/New Zealand, and Japan, where it is often the seventh most prevalent cancer, it ranks higher. Descriptive epidemiology databases frequently combine renal cell carcinomas with other upper urinary tract cancers for the sake of cross‐national comparability and cross‐temporal consistency. In Europe, kidney cancer claims 39,000 lives each year and accounts for around 99,000 new cases in 2018. In several other European Union (EU) regions, mortality is still trending upward, even if it has decreased over the past three decades in Northern and Western Europe (Huang et al., 2022).

Six groups were created from male wistar albino rats: group 2 (cisplatin control); group 1 (normal); groups mangiferin 10, 20, and 40 mg/kg in steps 3, 4, and 5; and group 6 (40 mg/kg). Ten days were dedicated to the treatment. A single 8 mg/kg dosage of cisplatin was given on day 7 to every group excluding normal and itself in order to cause nephrotoxicity. Day 11 saw the anesthesia of the animals, the extraction of cardiac blood, and the separation of serum. After that, kidneys were removed from the rats and stored for histological, ultrastructural, immunohistochemical, and western blot analyses. Increased oxidative stress and inflammation in the cisplatin control group led to a marked decrease in renal function, which was further supported by histopathology and the expression of MAPK pathway proteins (Du et al., 2020).

The study's goal was to investigate the in vitro dose‐dependent effects on mangiferin and cisplatin. Normal kidney epithelial (NkE) cells were subjected to cisplatin's cytotoxic effects using the MTT assay, cell viability at 25 μM concentration was established to be 50.7% higher than that of control cells. It was found that the vitality of the cells was 50.05% higher than control cells according to the dose succeeding a 24‐h period spent in cisplatin. After 2 h of mangiferin treatment, 20 μM was able to successfully counteract the poisonous impact on cisplatin. What morphology of the nucleus may change dramatically after being given a 25 μM cisplatin exposure for 24 h? Morphological alterations were significantly attenuated in the pretreatment cells treated with 20 μM mangiferin. It can be inferred that mangiferin was a potentially useful drug that may be used to lessen the nephrotoxic effect and produce a synergistic anticancer effect (Lum et al., 2022).

Numerous in vitro studies were directed about SKRC‐45 (a human renal cell cancer metastatic cell line) to study the healing effectiveness about cisplatin or mangiferin being present. Mangiferin and cisplatin were administered to the cells concurrently in a dose‐dependent fashion. Both bright field microscopy and the MTT cell viability assay were then utilized to calculate cytotoxicity. Additionally, solid tumor Ehrlich ascites carcinoma (EAC) models albino mice in Switzerland were created using those procedures previously explained in order to evaluate cisplatin's antitumor efficaciousness in experimental animals given mangiferin. The six‐week Swiss albino mice, an old male had his right flank subcutaneously injected with EAC cells. Ten days later, the animals were split into four groups at random of four each. The following were the groups: In charge (jellyfish with tumors not yet treated), depending on the type of tumor, mice were either: (a) given a mangiferin treatment (20 milligrams per kilogram body weight on alternate days in a row); (b) given a cis treatment (20 milligrams per kilogram body weight on alternate days for a span of 21 days and 10 mg/kg bw (milligrams per kilogram of body weight) once a week); or (c) given a combination of (cisplatin plus mangiferin for 21 days) for tumor‐bearing mice. Cisplatin was administered intraperitoneally to all experimental animals, while mangiferin was administered orally. At the end of the trial period, tumor growth was measured, and the volume was assessed using the ellipsoid volume formula and a Vernier caliper. Additionally, tumor weights were assessed concurrently (Mittal et al., 2020). Mangiferin was discovered to reduce oxidative damage induction and also increase pro‐survival signaling cascades controlled by Nrf‐2 through PI3K initiation, thereby mitigating cisplatin‐induced nephrotoxicity in vivo as well as in vitro. Furthermore, within EAC cell‐caused solid tumor bearing experimental mice and SKRC‐45 and MCF‐7 cancer cell lines, mangiferin and also cisplatin exhibited synergistic antitumor efficacy (Sahu et al., 2019).

## 13 EFFECT OF MANGIFERIN ON OTHER TYPES OF CANCER

Worldwide, pancreatic cancer is a major cause of death, yet there are few available treatments. Additionally, the pancreatic cancer cells’ development of medication resistance makes management even more challenging (Aslanian et al., 2020). Less than 5% of cases of pancreatic cancer are pancreatic neuroendocrine tumors, because they are treated differently from pancreatic adenocarcinoma and have different characteristics (Rawla et al., 2019). Currently, the only successful treatment is early‐stage surgical resection (Cai et al., 2021). The anticancer properties of mangiferin against gemcitabine‐resistant Mia‐PaCa2 pancreatic cancer cells were investigated. Pancreatic cancer cell line proliferation was related to Normal and Mia‐PaCa2 cell lines. The MTT test was employed to investigate HTERT‐PINE, and western blot and fluorescence microscopy were used to identify apoptosis. Using flow cytometry, the effects on the mitochondrial membrane potential (MMP), the cell cycle, and the generation of reactive oxygen species (ROS) were assessed. When combined with western blot, fluorescence microscopy proved that mangiferin activated autophagy. With an IC50 of 10 μM, mangiferin suppressed the development of the Mia‐PaCa2 cells. Normal cells were less vulnerable to mangiferin's harmful effects. Mangiferin initiated. In the Mia‐PaCa2 cells, apoptosis was connected to an upsurge in the Bax/Bcl‐2 percentage. It showed that mangiferin induced autophagy that was expressed by Mia‐PaCa2 cells and increased Beclin‐1 and LC3 II expressions. Mangiferin inhibited the capacity of the Mia‐PaCa2 cells to invade and migrate. It also caused cell‐cycle arrest and the formation of endogenous ROS. Mangiferin was established to block these actions (Yu et al., 2019).

In Western countries, oral cancer is an unusual cancer in a few world regions with high risk. It is mostly an avoidable tumor, because among the several risk factors acknowledged like alcohol consumption, tobacco use, and betel nut chewing (Garcia‐Martín et al., 2019). Finding possibly cancerous lesions on the oral mucosa and local factors that contribute to persistent inflammation are the first steps in prevention and diagnosis anticipation. As such, each lesion needs to be identified quickly and given the proper care. As much as 99% of oral cancers and premalignancies can be identified through the medical detection with examination of lesions on the mucosa in the mouth. Parietal and oral cancers combined are the sixth most prevalent cancer globally and are among the top three in regions with high prevalence. The estimated yearly incidence of pharyngeal cancers, excluding nasopharynx, is 130,300, with two thirds of cases occurring in underdeveloped nations. The expected incidence for mouth cancers is similar, at about 275,000. Oral cancer incidence varies significantly by region (about 20 times) (Shrestha et al., 2020). On hamster cheek pouch carcinoma (HCPC) induced by 0.5% DMBA, or 7.12‐dimethylbenz[a]anthracene, researchers evaluated the anticancer potential on mangiferin in terms of antioxidants, lipid peroxidation (LPO), and detoxification enzyme levels. Treatment with DMBA thrice a week for more than 14 days was the cause of oral cancer on the hamster buccal pouch (HBP). Regarding hamsters with DMBA‐challenged buccal pouch cancer (BPC), completely specified Oral cancer (OC) establishment using antioxidant, LPO, body weight (bw), tumor burden (TB), and liver marker enzymes were noted, along with histological alterations. When mangiferin was given orally to DMBA‐painted hamsters at a concentration of 50 g/kg bw, the animals showed significant reductions in body weight, tumor progression, biochemical alterations, and histological changes. The study's findings unequivocally show that mangiferin's anticarcinoma impact modulates strong agents for detoxification, anti‐LPO, and antioxidants to eliminate malignant cells’ metabolites on BPC in hamsters stimulated by DMBA (Liu et al., 2021).

The most common type of cancer in people is thyroid cancer. Consequently, a deeper comprehension of thyroid carcinogenesis and advancements in treatment are required. Geographical differences in the frequency of thyroid carcinoma are significant, particularly among women. The largest incidence was seen in higher‐income nations, such as the United States, the Republic of Korea, Croatia, Austria, Italy, France, and Israel and several intermediate‐ to upper‐middle‐earnings nations like China, Turkey, Brazil, and Costa Rica (Kitahara & Schneider, 2022). Additionally, a number of island states and territories, such as Puerto Rico, French Polynesia, New Caledonia, Cyprus, and Cabo Verde, have high incidence rates (Lam et al., 2022). Although environmental exposures may also be a factor, it is believed that geographic variations in availability to care and diagnostic procedures account for the majority of this variation (Doghish et al., 2023).

Researchers concentrated on examining how mangiferin affected TPC1, a human thyroid cancer cell line, and evaluating its potential as a treatment for aggressive thyroid tumors. By using the MTT technique, the TPC1 cells’ vitality was evaluated. The compound's effects on apoptosis were assessed using fluorescence microscopy and the 4′‐6‐diamidino‐2‐phenylindole (DAPI) and acridine orange (AO)/estrogen receptor (ER) staining. Researchers also looked at the proliferating cell nuclear antigen (PCNA) expression. Mangiferin dramatically reduces TPC1 cell proliferation in the current investigation. Mangiferin‐treated cells (4 μM) showed signs of apoptosis in their nuclei, as demonstrated by DAPI nuclear staining. TPC1 cell exposed to two and four μM mangiferin resulted in cell death, as demonstrated by AO/EtBr staining. Through the initiation of caspase‐3 and reduced expression of Bcl‐2, mangiferin promotes apoptosis. The apoptotic pathways were found to be activated by mangiferin. Additionally, by inhibiting PCNA, it reduced TPC1 cell survival (Zhang & Wang, 2018). The incidence of skin cancer is quickly increasing and is very prevalent. Melanoma death rates are rising while nonmelanoma skin cancer (NMSC) death rates are down. Significant morbidity is linked to both melanoma and NMSC. While severe, sporadic sun exposure appears to be associated with the development of melanoma, persistent sun exposure is the primary cause of nonmalignant skin cancer (NMSC). Melanoma and NMSC incidence rates are on the rise, which can be attributed in part to ozone depletion. Unlike NMSC, ultraviolet (UV) radiation and melanoma do not directly correlate. Melanoma risk throughout life is greatly increased by genetic predisposition (Leiter et al., 2020). One of the most severe skin cancers, advanced metastatic melanoma, presently has no effective treatment. For most solid cancers, including melanomas, to proliferate and metastasize, the angiogenesis process is essential (Dildar et al., 2021).

This prevents angiogenesis, which is a feature of the chorioallantoic membrane assay in chicken eggs and in in vivo syngeneic studies of melanoma, as well as capillary tubes, angiogenic, and invasive processes development of melanoma cells that have spread and in vitro human placental blood vessel explants. The study's conclusions demonstrated that the natural glucosylxanthone mangiferin has intriguing anti‐angiogenic properties that warrant additional (pre)clinical research in patients with melanoma cancer (Delgado‐Hernández et al., 2020). Despite tremendous advancements in the healing of many additional cancer types, brain tumors continue to be a major as well as unresolved therapeutic concern. As many as 18,500 of the projected 43,800 primary brain tumors that occur annually in the US are predicted to be malignant. At least 12,690 deaths in the US are currently attributed to brain tumors, which also happen to be the most frequent reason why children under the age of 0 die from cancer to 14 (Upton et al., 2022).

On U‐87 cell lines, cytotoxicity tests and flow cytometry were carried out. A spatiotemporal pattern of release at the pH of cancer cells was verified by drug release tests. Studies on cytotoxicity demonstrated a 1.28‐fold reduction in the IC50 value, signifying efficacious anticancer action, whereas the hemolytic profile demonstrated safety. Using flow cytometry, it was confirmed that the nanoconjugate effectively induced apoptosis with less necrosis in comparison to the naïve medication (Harsha et al., 2019).

Recent countries as a result of their elderly populations are facing a major problem with bone illnesses with infections, cancer, and osteoporosis. Though there are already medicines obtainable in the clinic to cure these illnesses, some of them cause serious problems because of the side effects. For example, because of chemotherapy effects on both healthy and diseased tissues, it is not very good at differentiating between them (Lefteh et al., 2022).

Researchers assessed the impact that mangiferin had on rats that had osteopenia brought on by ovariectomy. The animals were divided into three groups with ovariectomies (OVX‐C, which were OVX‐M10 as the controls; which were OVX‐M30 with mangiferin (10 mg/kg/day), and which were 30 mg/kg/day of mangiferin and one SHAM (sham)‐operated group). Mangiferin was administered for 6 months. Therefore, mangiferin exhibited an antiresorptive effect on bone metabolism and reduced the bone alterations (Rahmani et al., 2023). Table 1 shows the anticancer potential of mangiferin.

**TABLE 1:** Mangiferin against cancers.

Type of cancer	Study	Intervention	Result	Reference
Breast cancer	In vivo: xenograft mouse model using MCF‐7 cells (mammary carcinoma)	Mangiferin at 100 mg/kg (oral) for 72 h	Inhibition of the development of MCF‐7 breast cancer cells, with respect to time	Zhang et al. (2020)
Breast cancer	Within living organisms: Cytotoxic mangiferin's impact on MDA‐MB‐231 (xenograft mice)	Mangiferin at 100 milligrams per kilogram for 4 days	Dose‐responsive suppression of MDA‐MB‐231 and MCF‐7 growth tumor substance	Deng et al. (2018)
Colon cancer	In vivo: Albino rat colon carcinogenesis	0.1% mangiferin for 48 h	65%–85% decrease in colonic mucosal cell proliferation	Samadarsi et al. (2022)
Colon cancer	In vitro: Characterization of cytotoxic effect of mangiferin on HeLa cells	48 h of exposure to 10 μg/mL mangiferin	Significant reduction in tumor nodule formation	Lauricella et al. (2019)
Liver cancer	In vivo: Healthy Sprague–Dawley rats hepatocellular carcinoma	Mangiferin at 50 mg for 12 weeks	Significant reduction in tumor nodule formation	Yang et al. (2019)
Liver cancer	In vivo: Mangiferin's inhibitory impact on liver of mice	Mangiferin taking 5 milligrams per kilogram of body weight, taken orally for a period of six days	Reduced proliferation on liver cell within a dose‐ and in a time‐dependent way	Tao et al. (2023)
Gastric cancer	In vitro: Antiproliferative mangiferin's impact on SGC‐7901 cells	Applying mangiferin for 24, 48, and 72 h at 16.00, 8.63, and 4.79 μmol/L	Caused gastric cancer cells to undergo apoptosis by blocking PI3K/Akt pathways	Du et al. (2018)
Gastric cancer	In vivo: Gastroprotective effect of mangiferin on 4‐h rats ligated with pylorus	Mangiferin at 3, 10, and 30 milligrams per kilogram of body weight for 48 h	50% and 76% of gastric damage suppression	Chi et al. (2021)
Ovarian cancer	In vivo: Cytotoxic effects of mangiferin on OVCAR8 cell (xenograft mice model)	Mangiferin at (8, 16, or 20 μM) for 20 days	Mangiferin boosted the cytotoxic effects on ovarian cancer cells	Kuroki and Guntupalli (2020)
Ovarian cancer	In vivo: Nude ES‐2 mouse xenograft model	24 h of exposure of mangiferin at 5, 10, and 25 μM	Inhibited the development of ovarian epithelial carcinoma in a dose‐dependent way	Zeng et al. (2020)
Prostate cancer	In vitro: Effect of mangiferin on PC3 cells	Mangiferin at (0, 10, 20, and 40 μM) for 72 h	Significantly reduced Bcl‐2 expression levels and boosted apoptotic death	Sarfraz et al. (2023)
Prostate cancer	In vitro: Mangiferin's cytotoxic effect on LNCaP cancer cells	24 h of exposure to mangiferin at (15 mol/mL)	Significantly reduced raises miR‐182 expression and Bcl‐2 expression levels	Khoobchandani et al. (2021)
Blood cancer	In vitro: Human leukemia cells HL‐60	(50 μmol/L) of mangiferin for 24 h	Reduced the expansion of HL‐60 cells within a dose‐reliant way	Ayatollahi et al. (2019)
Blood cancer	In vivo: BALB/c mice	40, 80, and 20 days of 120 mg/kg of Mangiferin	The survival rate was dramatically increased by mangiferin at 40 and 120 mg/kg	Morozkina et al. (2021)
Lung cancer	In vivo: Healthy Swiss albino mouse‐male	Mangiferin (100 mg/kg at body weight) after 20 days	Inhibition of growth of lung cancer cells in a dose‐dependent way	Spampinato et al. (2022)
Lung cancer	In vivo: Swiss albino mice	Mangiferin administered orally (100 mg kg^−1^ b/w) for 18 weeks	Inhibition in the growth of lung cancer cells with chemoprotective effect in a dose‐dependent way	Razura‐Carmona et al. (2022)
Kidney cancer	In vivo: *Male wistar albino rats*	For 10 days, mangiferin at 10, 20, and 40 mg/kg	Significant reduction in tumor nodule formation	Du et al. (2020)
Kidney cancer	In vitro: Effect of dose dependency of mangiferin using Nike cells	25 μM of mangiferin for 24 h	Ameliorative effect of mangiferin by upregulating through PI3K activation, Nrf‐2 displays synergistic anticancer action	Lum et al. (2022)
Bone cancer	In vitro: Effect of mangiferin on Saos‐2 and U2OS cells	25, 50, 75, and 100 μM of mangiferin for 24 h	Reduced proliferation of osteosarcoma	Wen et al. (2020)
Bone cancer	In vivo: Inhibitory effect of mangiferin on rats	Mangiferin at 10 mg/kg/day	Antiresorptive effect of mangiferin reduced the bone alterations	Rahmani et al. (2023)
Brain cancer	In vivo: Mangiferin's impact on DOX‐induced Sprague–Dawley male rats	Mangiferin after 7 months at a time between 30 and 60 mg/kg bw	Reduced oxidative stress and inflammation had neuroprotective impact on brain damage	Morozkina et al. (2021)

## 14 CONCLUSION

Mangiferin is a bioactive ingredient obtained from mango tree (*Mangifera indica* L), a plant that has multiple uses in medicine. The possible benefits of mangiferin have been validated in cases of many cancers, including breast cancer, brain, lung, cervical, prostate cancers as well as blood cancer. Besides its anti‐inflammatory as well as antioxidant properties, mangiferin's mechanism of action is discovered as opposing cancerous cells via *intra‐vivo* and *over‐vitro* sample. Mangiferin is still untested, but it has the potential to be taken into consideration as a medication candidate for the treatment of cancer. It has powerful anti‐inflammatory and antioxidant properties that may account for the improvements in life quality seen in cancer patients. Although mangiferin has not demonstrated cytotoxicity against cancer cells, its effects on gene regulation and immunomodulation (mostly on NF‐κB) warrant consideration for cancer immune treatment. Furthermore, based on its apoptotic and also anti‐angiogenic properties interminably specific cancerous cell lines as well as a few findings from living organism trials conducted using animal models, mangiferin is an obvious contender for use in cancer treatments down the road. Mangiferin can be found in many various sections of several plants, and the elements that can affect its content include the genotype, variety, along with cultivar among the plant, as well as the climate, geographical location, and growth stage at harvest, as well as circumstances for postharvest storage and processing techniques. Allocating funds into investigate mangiferin's distribution in various origins of plants and the variables influencing focused attention would improve understanding of where to find mangiferin and how best to recover it for use in dietary supplements or nutraceuticals. In addition, by improving recovery efficiency and reducing losses, new developments including cutting‐edge food preparation technology used for mangiferin's separation and purification will lower the expenses for collecting mangiferin originating from various vegetations. These days, there are a lot of good reasons to use mangiferin as a nutraceutical or as the only ingredient in the creation of a functional food. It may be effective in promoting anti‐inflammatory, antibacterial, and antioxidant bioactivities as well as potentially preventing chronic illnesses like cancer and diabetes. However, before any useful uses can be made, new technologies must be developed to enhance the inadequate bioavailability and low solubility. Through the regulation of several cell‐signaling molecules, mangiferin has shown its promise as an anticancer agent in a variety of cancer types. Mangiferin's putative anticancer actions involve preventing the growth of cancer cells, causing oxidative stress, inflammation, and angiogenesis, as well as triggering cell‐cycle arrest and apoptosis. Additionally, it has been documented that etoposide, oxaliplatin, doxorubicin, cisplatin, and 5‐fluorouracil have synergistic effects with anticancer medications by improving the efficacies of anticancer drugs by altering several cell‐signaling mechanisms. Recent research demonstrates that mangiferin (MGF) can treat a variety of malignancies while having little to no negative impact on healthy cells. Through targeting interleukins, growth factors, genes, enzymes, and signaling pathways, it enhances apoptosis and suppresses tumor growth, proliferation, motility, and angiogenesis. To make other therapeutic medications more sensitive to the body, it can also be taken with them. Additionally, it can be administered in place of medications that tumors exhibit resistance to. Mangiferin is safe, but the dosage for each type of cancer should be adjusted based on body weight. Furthermore, further data are required to recognize and measure any mangiferin metabolic products in action that may be produced by the gastrointestinal microbiota either following first‐pass metabolism in the liver and that may have an impact on the mangiferin's biological activities. Furthermore, some progress is made in modifying mangiferin through chemical and physical means to improve its solubility, bioavailability, and ultimately its bioactivity. Recent developments in the search for new uses in relation to the bioactive qualities originating from mangiferin include the development of new procedures for its application in food chains, such as salification, complexion, nanoparticlization, and co‐administration with additional ingredients using natural or synthetic chemicals. While these technologies have demonstrated efficacy in augmenting the mangiferin's solubility and bioavailability, several impediments persist during the economically viable production of highly commercial goods that are bioavailable and soluble, intended as application like dietary supplements or health products made naturally. Numerous requests for patents on the work of mangiferin in a form that is nanoencapsulated have recently resulted from the remarkable success recently acquired in creating various mangiferin‐encapsulated nanocarriers. Although mangiferin has not been used extensively as a clinical treatment and not much information is information accessible about the clinical success of feeding trials involving humans, more research is necessary to understand the metabolism and pharmacokinetics of this drug. This knowledge is essential, prior to the recommendation of safe and effective doses of mangiferin for use in human nutrition supplements conversely nutraceuticals.

## AUTHOR CONTRIBUTIONS

**Nimra Irshad:** Writing – original draft (equal). **Hammad Naeem:** Writing – original draft (equal). **Muhammad Shahbaz:** Writing – original draft (equal). **Muhammad Imran:** Conceptualization (equal); resources (equal); validation (equal). **Ahmed Mujtaba:** Investigation (equal); visualization (equal). **Muzzamal Hussain:** Visualization (equal); writing – review and editing (equal). **Waleed Al Abdulmonem:** Writing – review and editing (equal). **Suliman A. Alsagaby:** Conceptualization (equal); resources (equal). **Tadesse Fenta Yehuala:** Data curation (equal); supervision (equal). **Mohamed A. Abdelgawad:** Writing – review and editing (equal). **Mohammed M. Ghoneim:** Investigation (equal); methodology (equal). **Ehab M. Mostafa:** Data curation (equal); validation (equal). **Samy Selim:** Data curation (equal); writing – original draft (equal). **Soad K. Al Jaouni:** Conceptualization (equal); project administration (equal); supervision (equal).

## CONFLICT OF INTEREST STATEMENT

The authors declare no conflict of interest.

## Data Availability

The data that support the findings of this study are available from the corresponding author upon reasonable request.
